# Incremental Diagnostic Value of Clinical Information for Large Language Models Across Multiple Organs: Retrospective Study

**DOI:** 10.2196/94904

**Published:** 2026-08-26

**Authors:** Jinqi Zhang, Xiaoyi Wang, Yanfeng Zhao, Xiaolin Cui, Liyun Zhao, Xinming Zhao, Hongmei Zhang, Zheng Zhu

**Affiliations:** 1 Department of Diagnostic Radiology, National Cancer Center/National Clinical Research Center for Cancer/Cancer Hospital, Chinese Academy of Medical Sciences & Peking Union Medical College, No. 17, Panjiayuan Nanli, Chaoyang District, Beijing, 100021, China, 86 13520102729; 2 Department of Radiology, Beijing Friendship Hospital, Capital Medical University, Beijing, China

**Keywords:** large language models, diagnostic accuracy, clinical history, radiology report, radiologist, ChatGPT, DeepSeek, Gemini, laboratory investigation

## Abstract

**Background:**

Although large language models (LLMs) have demonstrated the ability to generate the impression section from radiology findings automatically, the incremental diagnostic value of clinical information for these models remains unclear.

**Objective:**

This study aimed to evaluate the incremental diagnostic value of clinical information for LLMs and compare their performance with that of radiologists.

**Methods:**

This retrospective study included radiology reports from patients with histopathologically confirmed liver, lung, and breast diseases from 2 institutions between October 2021 and February 2025. We defined three progressive information input scenarios: (1) basic patient information and imaging findings, (2) scenario A plus chief complaint or clinical history, and (3) scenario B plus key laboratory results. Scenario-based data were input into 3 general-purpose LLMs (DeepSeek-R1, Gemini 2.5 Pro, and GPT-4o), generating 2709 entries. Diagnostic accuracy was assessed for both benign-malignant differentiation and disease diagnosis, with histopathology serving as the reference standard. Accuracy was compared among scenarios and against radiologist performance using the McNemar test, and *P* values were adjusted using the Holm-Bonferroni correction for multiple comparisons.

**Results:**

A total of 301 patients with pathologically confirmed diseases were included (mean age 53.5, SD 12.0 years; women: n=208, 69.1%). In the liver cohort, a numerical trend toward higher accuracy was observed in scenario C compared with scenario A across all 3 models (scenario C range: 72.3%‐76.2% vs scenario A range: 64.4%‐68.3%); these differences did not reach statistical significance after Holm-Bonferroni correction (all adjusted *P*>.99). Notably, the DeepSeek-R1 model in scenario C achieved the highest diagnostic accuracy (77/101, 76.2%), with no evidence of a difference compared with radiologists (82/101, 81.2%; adjusted *P*>.99). In contrast, results in the lung and breast cohorts were more heterogeneous. In the lung cohort, GPT-4o achieved its highest accuracy in scenario A for disease diagnosis (68/92, 73.9%), which exceeded its performance in scenario B (64/92, 69.6%) and scenario C (66/92, 71.7%), suggesting that additional clinical information did not confer a consistent benefit. Gemini 2.5 Pro in scenario B achieved the highest accuracy in this cohort (72/92, 78.3%); however, no statistically significant difference was found compared with radiologists (80/92, 87.0%; adjusted *P*=.25). In the breast cohort, DeepSeek-R1 achieved the numerically highest diagnostic accuracy in scenario A, and it decreased numerically with the addition of laboratory tests, although no significant difference was found between scenarios A and C (73/108, 67.6% vs 71/108, 65.7%; adjusted *P*>.99).

**Conclusions:**

While the addition of clinical information was associated with a numeric trend toward higher diagnostic accuracy overall, this trend was heterogeneous across models and disease types, and no statistically significant improvement was demonstrated after adjustment for multiple comparisons.

## Introduction

With the increasing prevalence of large language models (LLMs), their application in radiology has expanded, offering potential benefits to both radiologists and patients. Current applications include evaluating medical knowledge on board examinations [1-3], simplifying radiology reports to improve patient readability [4-6], addressing patient inquiries [7-10], and extracting data from radiology reports [11-15]. The process of generating a diagnostic report typically comprises two steps: (1) identifying radiologic findings and (2) synthesizing these findings with clinical context to formulate the final impression. Zhou et al [16] evaluated the feasibility of using GPT-4V (OpenAI) to detect radiologic findings on chest radiographs and reported limited diagnostic accuracy for this initial step. Conversely, GPT-4 (OpenAI) has demonstrated the potential to detect errors in radiology reports [17]. Furthermore, it can use logical reasoning to resolve discrepancies [18], thereby enhancing radiologists’ efficiency. Regarding the second step, LLMs have demonstrated the ability to generate the impression section from radiology findings automatically [19-21] and to propose differential diagnoses based on imaging findings [22]. Sun et al [19] noted that GPT-4’s capabilities in zero-shot impression generation from radiology report findings remained largely unexplored and conducted preliminary research. They compared impressions generated by radiologists and GPT-4 across multiple dimensions, demonstrating GPT-4’s proficiency in linguistic coherence. Building on this work, Zhang et al [20] conducted further research by constructing a specialized LLM for radiology through pre-training and fine-tuning, extending report impression generation to comprehensive imaging modalities and anatomical regions. They found that while medical-specific models, such as WiNGPT, performed well across most dimensions, they significantly underperformed in providing specific diagnoses. Mukherjee et al [23] found that LLMs exhibit a stronger dependence on text than on images. Recently, several studies have begun to explore the incremental diagnostic value of clinical information. For instance, evaluations using the “Diagnosis Please” quizzes from radiology and pediatric case descriptions have demonstrated that integrating imaging findings with clinical history significantly improves the diagnostic accuracy of models such as GPT-4 and Claude 3.5 Sonnet [24-26]. Similarly, incorporating clinical information into the process of generating impressions from brain magnetic resonance imaging (MRI) findings has markedly enhanced model performance [27].

Meanwhile, LLM technology is rapidly advancing from closed-source families such as GPT to high-performance open-source models and specialized architectures that support local deployment [28-30]. However, these previous studies have primarily relied on rigorously curated textbook quizzes, focused on specific subspecialties, or evaluated only a limited number of models.

Therefore, the purpose of this study was to systematically evaluate the diagnostic accuracy of 3 general-purpose LLMs (DeepSeek-R1 [DeepSeek], Gemini 2.5 Pro [Google], and GPT-4o) in assessing liver, lung, and breast diseases across 3 progressive clinical information input scenarios.

## Methods

### Ethical Considerations

The study protocol was reviewed and approved by the Ethics Committee of the Cancer Hospital of the Chinese Academy of Medical Sciences (approval 25/331‐5277). The Ethics Committee waived the requirement for informed consent, determining that the retrospective use of deidentified data posed no risk to patients. To ensure confidentiality, all patient identifiers were removed prior to analysis. No images of individual participants are included in this manuscript or its supplementary materials.

### Research Data

Data for this study were obtained from 2 hospitals. Radiology reports were retrieved from the hospitals’ radiological information systems from October 2021 to February 2025, and consecutive cases were collected and comprised 101 liver, 92 lung, and 108 breast cases. Inclusion criteria were as follows: patients (1) aged ≥18 years; (2) who underwent contrast-enhanced liver MRI, chest computed tomography (CT), or breast MRI for disease screening or diagnostic purposes; and (3) who underwent surgical resection with histopathologic confirmation. Exclusion criteria were as follows: (1) lack of relevant preoperative laboratory results and (2) incomplete radiologic findings or impression text. For patients with multiple preoperative examinations, only the most recent scan before surgical resection was selected to ensure the closest temporal proximity to the pathological diagnosis and to maintain the independence of the observations.

### Data Collection and Preparation

We collected patient radiology reports containing both findings and impression sections, along with clinical information such as demographics, chief complaints, and key laboratory test results obtained prior to histopathologic examination. Then, we defined three progressive information input scenarios: (1) basic patient information and imaging findings, (2) scenario A plus the chief complaint or clinical history (only the chief complaint was available for liver and lung cases), and (3) scenario B plus key laboratory test results relevant to each disease. All patient-identifying information was anonymized. We then populated the collected data according to these 3 scenarios and generated separate templates for each disease. To mimic the real-world clinical context, the original text (in Chinese) was kept unchanged during input. Examples of templates for different diseases are provided in Multimedia Appendix 1. All model queries were conducted via the public consumer web interfaces of the evaluated models. These interfaces do not support separate system-level instructions; all role definitions, task descriptions, and formatting constraints were therefore incorporated directly into the user input templates provided in the supplementary materials. All clinical text was manually deidentified prior to input, with patient names, medical record numbers, and institutional identifiers removed. The option to decline data use for model training was selected in the data management settings. No content beyond the deidentified text shown in the appendix templates was entered.

It is important to note that while the LLMs were tested across 3 progressive experimental scenarios to evaluate the incremental value of additional data, the radiologist’s performance remained a fixed historical benchmark and was not retested under the same constrained scenarios.

All radiology reports used as the historical baseline were authored by experienced attending radiologists specializing in abdominal, thoracic, or breast imaging, and detailed demographic and experience characteristics are provided in Multimedia Appendix 2.

All radiology reports and clinical data used in this study were sourced exclusively from the institutional hospital information system with strictly controlled access. None of these records were publicly released or indexed at any time, and therefore, they could not have been included in the pre-training data of any evaluated model.

To minimize bias arising from the models’ context sensitivity and to avoid memory interference, chat sessions were restarted for each model, and responses were collected after each report was typed, yielding a total of 2709 entries.

### Report Processing and Evaluation

The LLM-generated reports were exported to Microsoft Excel. A binary semantic matching rubric was used to compare model outputs against the histopathologic reference standard. A case was deemed consistent (score 1) if the core diagnosis matched the reference, regardless of minor stylistic variation; it was deemed inconsistent (score 0) if the diagnosis contradicted the reference or no definitive diagnosis was given. For all models, only the final diagnostic output was evaluated; the internal reasoning traces generated by DeepSeek-R1 were not considered in the scoring process. All cases were independently evaluated by 2 raters: a radiologist (XW, with 23 y of experience) and a medical student (JZ, with 2 y of experience). Disagreements were resolved through consensus with a third evaluator, a radiologist (ZZ, with 21 y of experience). To assess the reliability of the free-text evaluation, interrater agreement between the 2 primary evaluators was measured using Cohen κ coefficient. The κ value and its 95% CI are reported in Table S1 in Multimedia Appendix 3.

For evaluation of the breast cohort, reports that included a specific diagnostic name were classified according to the established biological nature of that entity, irrespective of the Breast Imaging Reporting and Data System (BI-RADS) score. For reports without a specific diagnosis, a strict BI-RADS–based threshold was applied: categories 1 to 3 were defined as benign, and categories 4a to 6 were defined as malignant. For the purpose of determining diagnostic consistency, cases were classified as inconsistent if the original radiology report provided only a risk classification without explicitly stating a specific histologic diagnosis in the impression section. Per the classification rubric described earlier, any case deemed inconsistent was counted as an incorrect diagnosis and was not excluded from the denominator.

### LLMs

We used the 3 generic LLM versions GPT-4o (accessed July 11 to August 9, 2025), Gemini 2.5 Pro (accessed July 17 to August 9, 2025), and DeepSeek-R1 (accessed July 13-31, 2025). All model queries were performed via the public consumer web interfaces of the evaluated models. No specific hyperparameter values were manually set or configured during this process. For reference, the default hyperparameter values reported in the official documentation for each model are listed in Multimedia Appendix 2. The LLMs were tasked with generating radiologic impressions from the input data defined in the various scenarios.

### Study Design

We compared the diagnostic accuracy of LLMs with that of radiologists for “benign-malignant differentiation” and “disease diagnosis.” This evaluation was conducted using two reference standards: (1) for the entire study cohort, histopathologic diagnosis served as the reference standard; and (2) for the breast cohort specifically, the final impressions served as the reference standard to evaluate model performance in BI-RADS classification. We separately recorded the diagnostic accuracy of each model for the benign and malignant subgroups. Furthermore, we adhered to the principle of single-variable analysis to independently assess the effects of model architecture, input scenario, and disease type on diagnostic performance.

### Statistical Analyses

Data were analyzed using R software (version 4.4.2; R Foundation for Statistical Computing). To address the imbalanced nature of the cohorts, diagnostic performance for benign-malignant differentiation was evaluated comprehensively using overall accuracy, sensitivity, specificity, positive predictive value, and negative predictive value. Pairwise comparisons of diagnostic accuracy were performed using the McNemar test. To control the cumulative risk of type I errors (false positives) arising from multiple pairwise comparisons, the Holm-Bonferroni correction was applied to all *P* values derived from the McNemar tests. The correction was applied separately within each diagnostic task for each organ cohort, so that the family-wise error rate was controlled independently for each analysis unit. Statistical significance was defined as an adjusted *P*<.05.

## Results

### Study Sample

**Figure 1.**
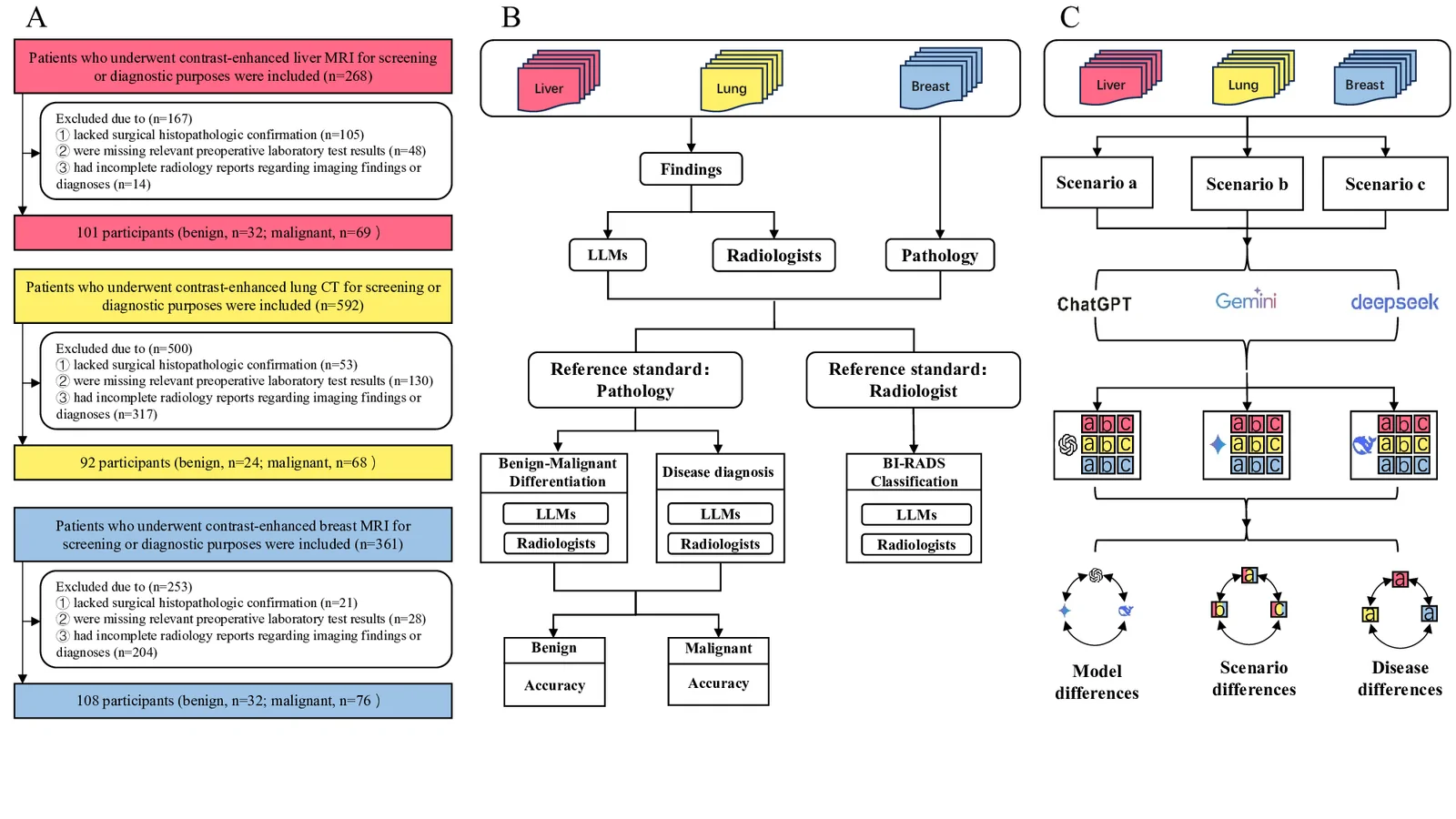
Study flowcharts. (A) Flowchart of patient inclusion and exclusion. (B, C) Study design workflow. We defined 3 progressive information input scenarios: A, basic patient information and imaging findings; B, scenario A plus chief complaint or clinical history; and C, scenario B plus key laboratory results. BI-RADS: Breast Imaging Reporting and Data System; ChatGPT: GPT-4o; CT: computed tomography; DeepSeek: DeepSeek-R1; Gemini: Gemini 2.5 Pro; LLM: large language model; MRI: magnetic resonance imaging.

A total of 301 patients with pathologically confirmed diseases were included in the study (liver: n=101, 33.5%; lung: n=92, 30.6%; and breast: n=108, 35.9%). The flowchart of patient inclusion and exclusion and the diagram of the study design are shown in Figure 1. The mean patient age was 55.1 (SD 12.2) years for the liver cohort, 54.5 (SD 12.3) years for the lung cohort, and 51.1 (SD 11.4) years for the breast cohort. There were 38 (37.6%), 62 (67.4%), and 108 (100.0%) female patients in the liver, lung, and breast cohorts, respectively. Detailed patient demographics and pathologic characteristics are summarized in Table 1.

**Table 1.** Patient demographics and histopathologic reference standards.

Characteristic	Liver (n=101)	Lung (n=92)	Breast (n=108)
Age (y), mean (SD)	55.1 (12.2)	54.5 (12.3)	51.1 (11.4)
Sex, n (%)			
Female	38 (37.6)	62 (67.4)	108 (100.0)
Male	63 (62.4)	30 (32.6)	0 (0.0)
Lesion characterization, n (%)			
Benign	32 (31.7)	24 (26.1)	32 (29.6)
Malignant	69 (68.3)	68 (73.9)	76 (70.4)
Histopathologic diagnosis, n (%)			
HCC^a^	48 (47.5)	—^b^	—
LUAD^c^	—	66 (71.7)	—
IBC^d^	—	—	68 (63.0)
Hemangioma	14 (13.9)	—	—
PSP^e^	—	14 (15.2)	—
FA^f^	—	—	21 (19.4)
Other^g^	39 (38.6)	—	—
Other^h^	—	12 (13.0)	—
Other^i^	—	—	19 (17.6)

a HCC: hepatocellular carcinoma.

b Not applicable.

c LUAD: lung adenocarcinoma.

d IBC: invasive breast carcinoma.

e PSP: pulmonary sclerosing pneumocytoma.

f FA: fibroadenoma.

g Other liver pathologies include cholangiocarcinoma, liver metastasis, hepatic adenoma, focal nodular hyperplasia, angiomyolipoma, low-grade intraepithelial neoplasia, pleomorphic undifferentiated sarcoma, hepatic perivascular epithelioid cell tumor, combined hepatocellular cholangiocarcinoma, and epithelioid hemangioendothelioma.

h Other lung pathologies include squamous cell carcinoma, atypical adenomatous hyperplasia, pulmonary lymphangioma, and bronchiolar adenoma.

i Other breast pathologies include ductal carcinoma in situ, intraductal papilloma, lipoma, adenomyoma, and malignant phyllodes tumor.

### Comparison of Diagnostic Accuracy in Different Scenarios

#### Overview

**Figure 2.**
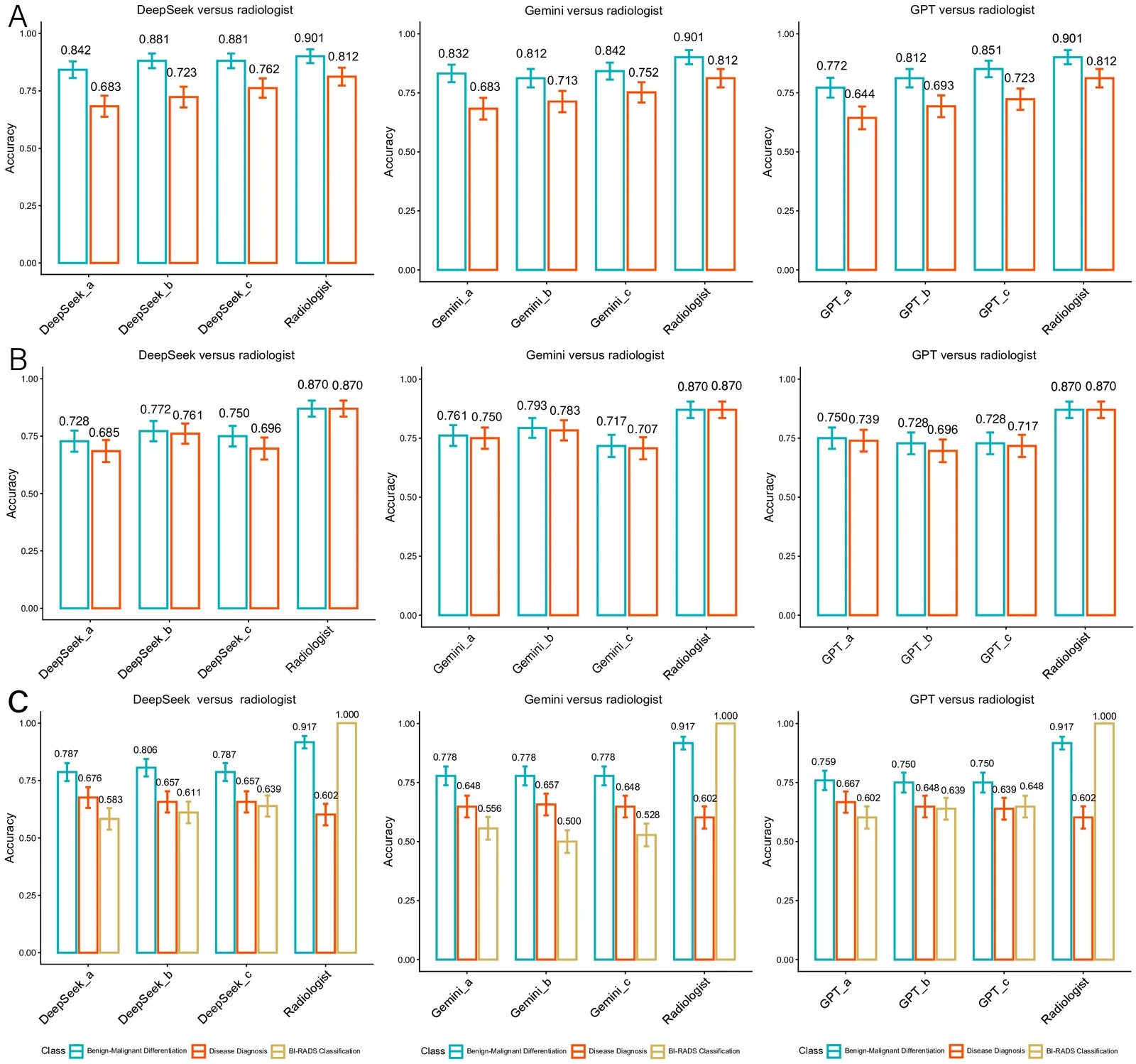
Impact of clinical information on diagnostic accuracy. Bar graphs illustrate the diagnostic accuracy across 3 progressive input scenarios in the (A) liver, (B) lung, and (C) breast cohorts. Performance is evaluated for benign-malignant differentiation, disease diagnosis, and Breast Imaging Reporting and Data System (BI-RADS) classification. We defined 3 progressive information input scenarios: A, basic patient information and imaging findings; B, scenario A plus chief complaint or clinical history; and C, scenario B plus key laboratory results. DeepSeek: DeepSeek-R1; Gemini: Gemini 2.5 Pro; GPT: GPT-4o.

The accuracy of the models for benign-malignant differentiation and disease diagnosis across the 3 scenarios is presented in Tables 2 and 3 and Figure 2. Overall, the 3 LLMs demonstrated consistent performance trends across liver, lung, and breast diseases. Diagnostic accuracy was higher for benign-malignant differentiation (range 71.7%‐88.1%) than for disease diagnosis (range 50%‐78.7%). Given the consistency of these trends, the subsequent results focus on disease diagnosis. All diagnostic performance metric values are presented in Table S2 in Multimedia Appendix 3. Additionally, we performed McNemar tests for all combinations of models and scenarios; the *P* value matrices for all pairwise comparisons are provided in Table S3 in Multimedia Appendix 3.

**Table 2.** Diagnostic accuracy of large language models (LLMs) versus radiologists for benign-malignant differentiation^a^.

Organ and scenario	Radiologist		DeepSeek^b^		Gemini^c^		GPT^d^		*P* value^e^		
n (%)	95% CI	n (%)	95% CI	n (%)	95% CI	n (%)	95% CI	DeepSeek versus radiologist	Gemini versus radiologist	GPT versus radiologist
Liver (n=101)											
a^f^	91 (90.1)	82.1‐94.9	85 (84.2)	75.2‐90.4	84 (83.2)	74.1‐89.6	78 (77.2)	67.6‐84.7	.15	.10	.002
b^g^	91 (90.1)	82.1‐94.9	89 (88.1)	79.8‐93.4	82 (81.2)	71.9‐88.0	82 (81.2)	71.9‐88.0	.75	.02	.03
c^h^	91 (90.1)	82.1‐94.9	89 (88.1)	79.8‐93.4	85 (84.2)	75.2‐90.4	86 (85.1)	76.4‐91.2	.75	.11	.27
Lung (n=92)											
a	80 (87.0)	77.9‐92.8	67 (72.8)	62.4‐81.3	70 (76.1)	65.9‐84.1	69 (75.0)	64.7‐83.2	<.001^i^	.004	.003
b	80 (87.0)	77.9‐92.8	71 (77.2)	67.0‐85.0	73 (79.3)	69.4‐86.8	67 (72.8)	62.4‐81.3	.008	.02	<.001^i^
c	80 (87.0)	77.9‐92.8	69 (75.0)	64.7‐83.2	66 (71.7)	61.2‐80.4	67 (72.8)	62.4‐81.3	.01	<.001^i^	<.001^i^
Breast (n=108)											
a	99 (91.7)	84.4‐95.9	85 (78.7)	69.6‐85.8	84 (77.8)	68.6‐85.0	82 (75.9)	66.6‐83.4	.006	.004	.001^i^
b	99 (91.7)	84.4‐95.9	87 (80.6)	71.6‐87.3	84 (77.8)	68.6‐85.0	81 (75.0)	65.6‐82.6	.01	.004	<.001^i^
c	99 (91.7)	84.4‐95.9	85 (78.7)	69.6‐85.8	84 (77.8)	68.6‐85.0	81 (75.0)	65.6‐82.6	.004	.004	<.001^i^

a Comparisons between LLMs and radiologists were made using the McNemar test.

b DeepSeek: DeepSeek-R1.

c Gemini: Gemini 2.5 Pro.

d GPT: GPT-4o.

e *P* values displayed are nominal (unadjusted).

f Basic patient information and imaging findings.

g Scenario A plus chief complaint or clinical history.

h Scenario B plus key laboratory results.

i Effects that remained significant after Holm-Bonferroni correction

**Table 3.** Diagnostic accuracy of large language models (LLMs) versus radiologists for disease diagnosis^a^.

Organ and scenario	Radiologist		DeepSeek^b^		Gemini^c^		GPT^d^		*P* value^e^		
	n (%)	95% CI	n (%)	95% CI	n (%)	95% CI	n (%)	95% CI	DeepSeek versus radiologist	Gemini versus radiologist	GPT versus radiologist
Liver (n=101)											
a^f^	82 (81.2)	71.9‐88.0	69 (68.3)	58.2‐77.0	69 (68.3)	58.2‐77.0	65 (64.4)	54.1‐73.5	.004	.004	<.001^g^
b^h^	82 (81.2)	71.9‐88.0	73 (72.3)	62.3‐80.5	72 (71.3)	61.3‐79.6	70 (69.3)	59.2‐77.9	.02	.009	.003
c^i^	82 (81.2)	71.9‐88.0	77 (76.2)	66.5‐83.9	76 (75.2)	65.5‐83.1	73 (72.3)	62.3‐80.5	.18	.08	.07
Lung (n=92)											
a	80 (87.0)	77.9‐92.8	63 (68.5)	57.8‐77.5	69 (75.0)	64.7‐83.2	68 (73.9)	63.5‐82.3	<.001^g^	.003	.002^g^
b	80 (87.0)	77.9‐92.8	70 (76.1)	65.9‐84.1	72 (78.3)	68.2‐85.9	64 (69.6)	59.0‐78.5	.004	.01	<.001^g^
c	80 (87.0)	77.9‐92.8	64 (69.6)	59.0‐78.5	65 (70.7)	60.1‐79.5	66 (71.7)	61.2‐80.4	<.001^g^	<.001^g^	<.001^g^
Breast (n=108)											
a	65 (60.2)	50.3‐69.3	73 (67.6)	57.8‐76.1	70 (64.8)	55.0‐73.6	72 (66.7)	56.9‐75.3	.24	.53	.36
b	65 (60.2)	50.3‐69.3	71 (65.7)	55.9‐74.4	71 (65.7)	55.9‐74.4	70 (64.8)	55.0‐73.6	.42	.43	.56
c	65 (60.2)	50.3‐69.3	71 (65.7)	55.9‐74.4	70 (64.8)	55.0‐73.6	69 (63.9)	54.0‐72.7	.42	.50	.65
BI-RADS^j^ (n=108)											
a	108 (100.0)	95.7‐100.0	63 (58.3)	48.4‐67.6	60 (55.6)	45.7‐65.0	65 (60.2)	50.3‐69.3	NA^k^	NA	NA
b	108 (100.0)	95.7‐100.0	66 (61.1)	51.2‐70.2	54 (50.0)	40.7‐59.3	69 (63.9)	54.0‐72.7	NA	NA	NA
c	108 (100.0)	95.7‐100.0	69 (63.9)	54.0‐72.7	57 (52.8)	43.0‐62.4	70 (64.8)	55.0‐73.6	NA	NA	NA

a Comparisons between LLMs and radiologists were made using the McNemar test.

b DeepSeek: DeepSeek-R1.

c Gemini: Gemini 2.5 Pro.

d GPT: GPT-4o.

e *P* values displayed are nominal (unadjusted).

f Basic patient information and imaging findings.

g Effects that remained significant after Holm-Bonferroni correction.

h Scenario A plus chief complaint or clinical history.

i Scenario B plus key laboratory results.

j BI-RADS: Breast Imaging Reporting and Data System.

k NA: not applicable.

#### Liver Cohort

As shown in Figure 2A, the diagnostic accuracy in scenario C was higher than in scenario A for DeepSeek-R1 (77/101, 76.2%, vs 69/101, 68.3%), Gemini 2.5 Pro (76/101, 75.2%, vs 69/101, 68.3%), and GPT-4o (73/101, 72.3%, vs 65/101, 64.4%). No significant difference was observed between the scenarios (all adjusted *P*>.99).

#### Lung Cohort

As shown in Figure 2B, the DeepSeek-R1 and Gemini 2.5 Pro models showed numerically higher diagnostic accuracy in scenario B than in scenario A, without reaching statistical significance (DeepSeek-R1: 70/92, 76.1%, vs 63/92, 68.5%; Gemini 2.5 Pro: 72/92, 78.3%, vs 69/92, 75.0%; all adjusted *P*>.99). In contrast, GPT-4o achieved its highest numerical accuracy in scenario A (68/92, 73.9%), which was not significantly different from scenario B (64/92, 69.6%) or scenario C (66/92, 71.7%; all adjusted *P*>.99).

#### Breast Cohort

As shown in Figure 2C, the diagnostic accuracy of DeepSeek-R1 was numerically highest in scenario A. It decreased numerically with the addition of laboratory tests (scenario C), although no significant difference was found between scenarios A and C (73/108, 67.6%, vs 71/108, 65.7%; adjusted *P*>.99). For GPT-4o, diagnostic accuracy was numerically highest in scenario A (72/108, 66.7%), with no evidence of a significant difference compared with scenario B (70/108, 64.8%) or scenario C (69/108, 63.9%; all adjusted *P*>.99).

### Comparison of the Diagnostic Accuracy in Different Models

**Figure 3.**
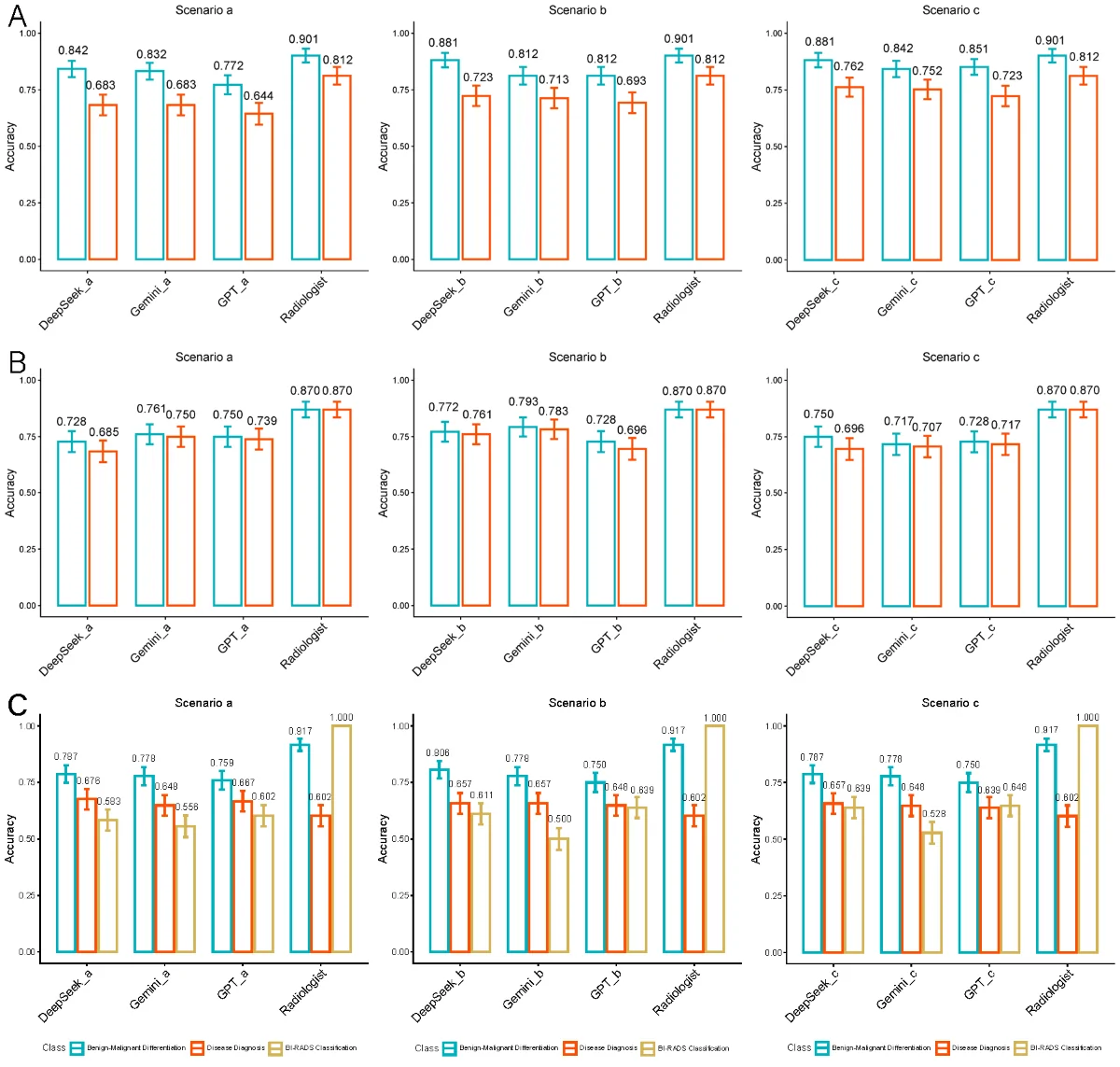
Comparison of diagnostic accuracy among 3 large language models (LLMs). Bar graphs compare the performance of LLMs within the same input scenarios for the (A) liver, (B) lung, and (C) breast cohorts. Metrics include benign-malignant differentiation, disease diagnosis, and Breast Imaging Reporting and Data System (BI-RADS) classification. We defined 3 progressive information input scenarios: A, basic patient information and imaging findings; B, scenario A plus chief complaint or clinical history; and C, scenario B plus key laboratory results. DeepSeek: DeepSeek-R1; Gemini: Gemini 2.5 Pro; GPT: GPT-4o.

As shown in Figure 3, the diagnostic performance of the 3 LLMs was compared with that of radiologists within identical input scenarios.

#### Liver Cohort

As shown in Figure 3A, radiologists achieved the highest diagnostic accuracy for both benign-malignant differentiation (91/101, 90.1%) and disease diagnosis (82/101, 81.2%). Among the 3 models, DeepSeek-R1 demonstrated the highest accuracy. Regarding benign-malignant differentiation, no evidence of a significant difference was found between DeepSeek-R1 (scenario A: 85/101, 84.2%; scenarios B and C: 89/101, 88.1%) and radiologists across all scenarios (all adjusted *P*>.99). Regarding disease diagnosis, the accuracy of the DeepSeek-R1 was 68.3% (69/101) in scenario A, 72.3% (73/101) in scenario B, and 76.2% (77/101) in scenario C. No statistically significant difference was found between the DeepSeek-R1 and radiologists in any scenario after Holm-Bonferroni correction, although numerical trends were noted in scenario A (unadjusted *P*=.004; adjusted *P*=.09) and scenario B (unadjusted *P*=.02; adjusted *P*=.35). In scenario C, no evidence of a difference was observed (adjusted *P*>.99).

#### Lung Cohort

As shown in Figure 3B, radiologists achieved the highest diagnostic accuracy for both benign-malignant differentiation and disease diagnosis (80/92, 87.0% for both). In scenarios A and B, the Gemini 2.5 Pro demonstrated the highest accuracy among the 3 models. In the unadjusted analyses, its accuracy was significantly lower than that of the radiologists for both benign-malignant differentiation (scenario A: 70/92, 76.1%; scenario B: 73/92, 79.3%; all unadjusted *P*<.05) and disease diagnosis (scenario A: 69/92, 75.0%; scenario B: 72/92, 78.3%; all unadjusted *P*<.05); however, these differences did not reach statistical significance after Holm-Bonferroni correction (all adjusted *P*>.05). In scenario C, regarding benign-malignant differentiation, the DeepSeek-R1 achieved the highest accuracy among the models (69/92, 75.0%), but no statistically significant difference was found between DeepSeek-R1 and radiologists (adjusted *P*=.20). Regarding disease diagnosis, the GPT-4o achieved the highest accuracy (66/92, 71.7%), which was also significantly lower than that of radiologists (adjusted *P*=.01).

#### Breast Cohort

As shown in Figure 3C, regarding benign-malignant differentiation, DeepSeek-R1 achieved the highest accuracy among the 3 models across all scenarios (scenarios A and C: 85/108, 78.7%; scenario B: 87/108, 80.6%). In the unadjusted analyses, its accuracy was significantly lower than that of radiologists (99/108, 91.7%) in all scenarios (all unadjusted *P*<.05); however, these differences did not reach statistical significance after Holm-Bonferroni correction (all adjusted *P*>.05). Regarding disease diagnosis, DeepSeek-R1 also demonstrated the highest accuracy among the models (scenario A: 73/108, 67.6%; scenarios B and C: 71/108, 65.7%). Although the accuracy was numerically higher than that of radiologists (65/108, 60.2%), no evidence of a significant difference was found (all adjusted *P*>.99; Tables 2 and 3).

### Comparison of Diagnostic Accuracy in Different Diseases

**Figure 4.**
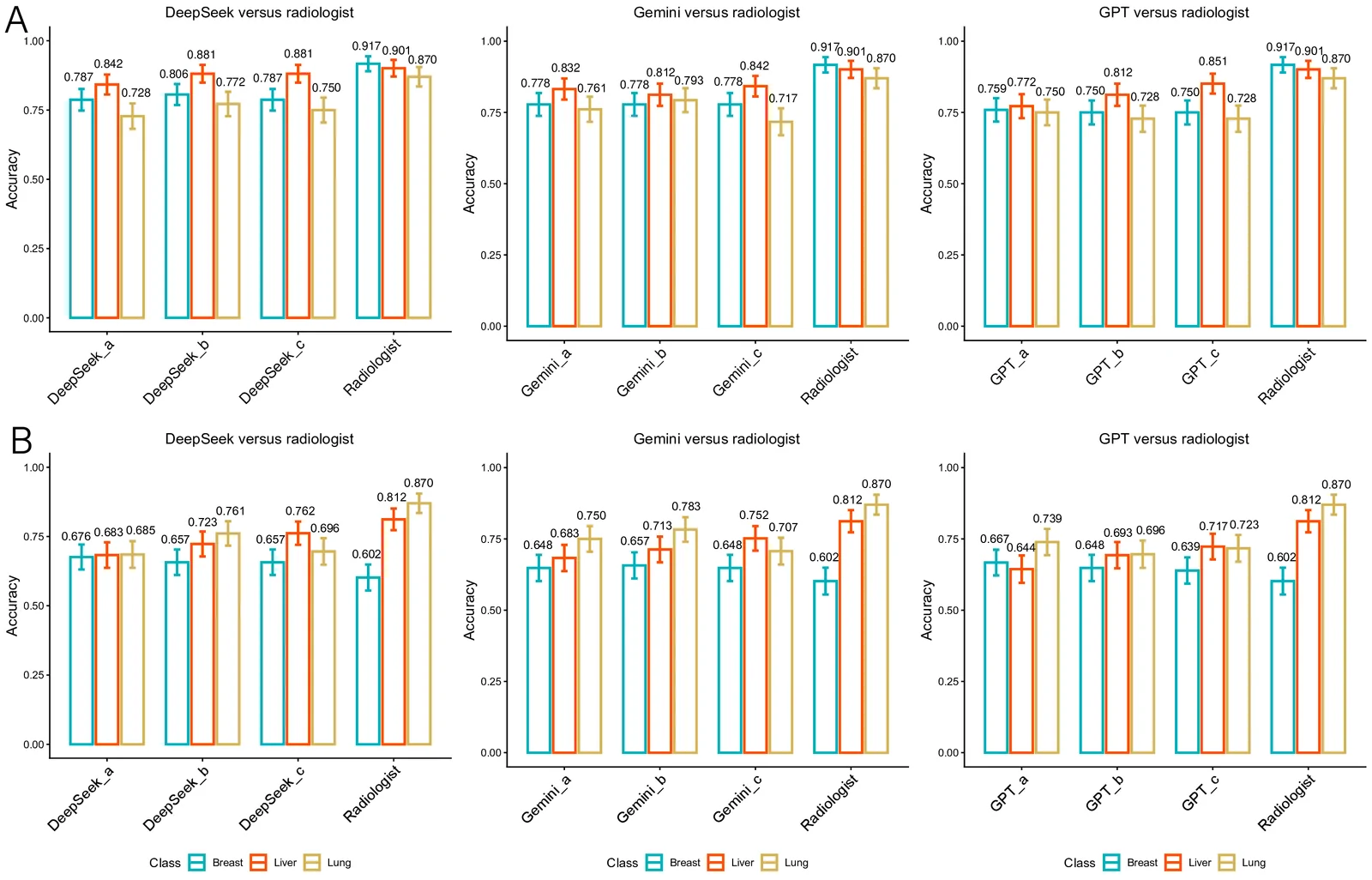
Bar graphs illustrate accuracy for (A) benign-malignant differentiation and (B) disease diagnosis. Results are shown for the liver, lung, and breast cohorts across 3 input scenarios for each model. We defined 3 progressive information input scenarios: A, basic patient information and imaging findings; B, scenario A plus chief complaint or clinical history; and C, scenario B plus key laboratory results. DeepSeek: DeepSeek-R1; Gemini: Gemini 2.5 Pro; GPT: GPT-4o.

Regarding benign-malignant differentiation, the 3 LLMs consistently achieved their highest accuracy in the liver lesions across all scenarios (range 77.2%‐88.1%; Table 2 and Figure 4A). Regarding disease diagnosis, the lowest accuracy was observed in the breast cohort across all models and scenarios (range 63.9%‐67.6%; Table 3 and Figure 4B).

### Diagnostic Accuracy of Benign and Malignant Subgroups

**Figure 5.**
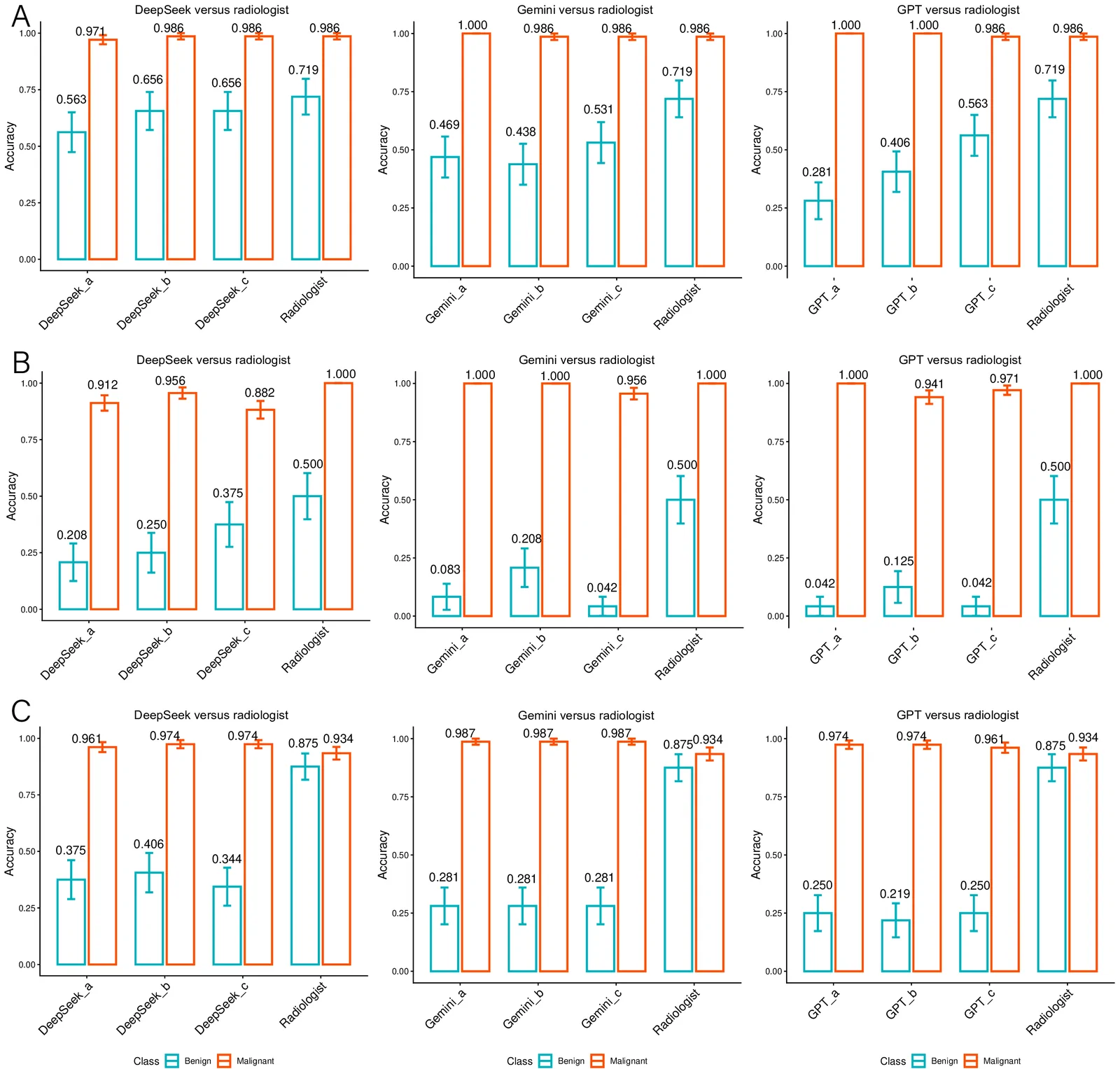
Subgroup analysis for benign-malignant differentiation. Diagnostic accuracy of 3 large language models is stratified by benign and malignant lesion status in the (A) liver, (B) lung, and (C) breast cohorts. We defined 3 progressive information input scenarios: A, basic patient information and imaging findings; B, scenario A plus chief complaint or clinical history; and C, scenario B plus key laboratory results. DeepSeek: DeepSeek-R1; Gemini: Gemini 2.5 Pro; GPT: GPT-4o.

**Figure 6.**
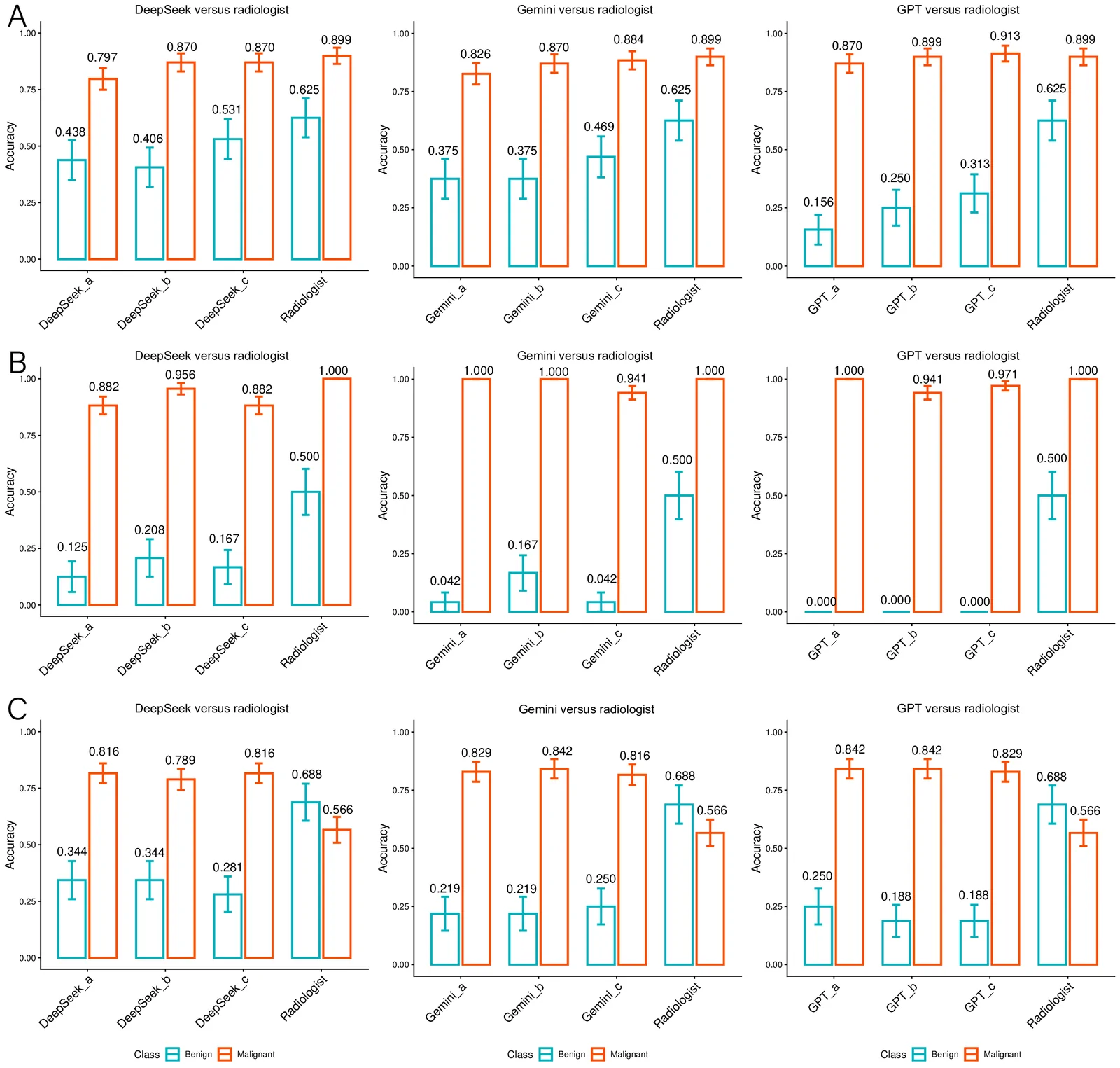
Subgroup analysis for disease diagnosis. Diagnostic accuracy of 3 large language models is stratified by benign and malignant lesion status in the (A) liver, (B) lung, and (C) breast cohorts. We defined 3 progressive information input scenarios: A, basic patient information and imaging findings; B, scenario A plus chief complaint or clinical history; and C, scenario B plus key laboratory results. DeepSeek: DeepSeek-R1; Gemini: Gemini 2.5 Pro; GPT: GPT-4o.

Diagnostic accuracy and 95% CIs for all cases, stratified by benign and malignant status, are summarized in Table 4. Figures 5 and 6 illustrate model accuracy across these subgroups. Overall, for both benign-malignant differentiation and disease diagnosis, the LLMs demonstrated higher accuracy for malignant lesions (benign-malignant differentiation: range 88.2%‐100%; and disease diagnosis: range 78.9%‐100%) than for benign lesions (benign-malignant differentiation: range 4.2%‐65.6%; disease diagnosis: range 0%‐53.1%). Notably, in the lung cohort diagnosis, GPT-4o achieved 0% accuracy for benign lesions across all 3 scenarios.

**Table 4.** Diagnostic accuracy of large language models stratified by benign and malignant.

Parameter	Benign, % (95% CI)			Malignant, % (95% CI)		
Liver (n=101)	Lung (n=92)	Breast (n=108)	Liver (n=101)	Lung (n=92)	Breast (n=108)
Differentiation^a^						
Radiologist	71.9 (53.0‐85.6)	50.0 (31.4‐68.6)	87.5 (70.1‐95.9)	98.6 (91.1‐99.9)	100.0 (93.3‐100.0)	93.4 (84.7‐97.6)
DeepSeek^b^-a^c^	56.2 (37.9‐73.2)	20.8 (7.9‐42.7)	37.5 (21.7‐56.3)	97.1 (89.0‐99.5)	91.2 (81.1‐96.4)	96.1 (88.1‐99.0)
DeepSeek-b^d^	65.6 (46.8‐80.8)	25.0 (10.6‐47.1)	40.6 (24.2‐59.2)	98.6 (91.1‐99.9)	95.6 (86.8‐98.9)	97.4 (90.0‐99.5)
DeepSeek-c^e^	65.6 (46.8‐80.8)	37.5 (19.6‐59.2)	34.4 (19.2‐53.2)	98.6 (91.1‐99.9)	88.2 (77.6‐94.4)	97.4 (90.0‐99.5)
Gemini^f^-a	46.9 (29.5‐65.0)	8.3 (1.5‐28.5)	28.1 (14.4‐47.0)	100.0 (93.4‐100.0)	100.0 (93.3‐100.0)	98.7 (91.9‐99.9)
Gemini-b	43.8 (26.8‐62.1)	20.8 (7.9‐42.7)	28.1 (14.4‐47.0)	98.6 (91.1‐99.9)	100.0 (93.3‐100.0)	98.7 (91.9‐99.9)
Gemini-c	53.1 (35.0‐70.5)	4.2 (0.2‐23.1)	28.1 (14.4‐47.0)	98.6 (91.1‐99.9)	95.6 (86.8‐98.9)	98.7 (91.9‐99.9)
GPT^g^-a	28.1 (14.4‐47.0)	4.2 (0.2‐23.1)	25.0 (12.1‐43.8)	100.0 (93.4‐100.0)	100.0 (93.3‐100.0)	97.4 (90.0‐99.5)
GPT-b	40.6 (24.2‐59.2)	12.5 (3.3‐33.5)	21.9 (9.9‐40.4)	100.0 (93.4‐100.0)	94.1 (84.9‐98.1)	97.4 (90.0‐99.5)
GPT-c	56.2 (37.9‐73.2)	4.2 (0.2‐23.1)	25.0 (12.1‐43.8)	98.6 (91.1‐99.9)	97.1 (88.8‐99.5)	96.1 (88.1‐99.0)
Diagnosis^h^						
Radiologist	62.5 (43.7‐78.3)	50.0 (31.4‐68.6)	68.8 (49.9‐83.3)	89.9 (79.6‐95.5)	100.0 (93.3‐100.0)	56.6 (44.7‐67.7)
DeepSeek-a	43.8 (26.8‐62.1)	12.5 (3.3‐33.5)	34.4 (19.2‐53.2)	79.7 (68.0‐88.1)	88.2 (77.6‐94.4)	81.6 (70.7‐89.2)
DeepSeek-b	40.6 (24.2‐59.2)	20.8 (7.9‐42.7)	34.4 (19.2‐53.2)	87.0 (76.2‐93.5)	95.6 (86.8‐98.9)	78.9 (67.8‐87.1)
DeepSeek-c	53.1 (35.0‐70.5)	16.7 (5.5‐38.2)	28.1 (14.4‐47.0)	87.0 (76.2‐93.5)	88.2 (77.6‐94.4)	81.6 (70.7‐89.2)
Gemini-a	37.5 (21.7‐56.3)	4.2 (0.2‐23.1)	21.9 (9.9‐40.4)	82.6 (71.2‐90.3)	100.0 (93.3‐100.0)	82.9 (72.2‐90.2)
Gemini-b	37.5 (21.7‐56.3)	16.7 (5.5‐38.2)	21.9 (9.9‐40.4)	87.0 (76.2‐93.5)	100.0 (93.3‐100.0)	84.2 (73.6‐91.2)
Gemini-c	46.9 (29.5‐65.0)	4.2 (0.2‐23.1)	25.0 (12.1‐43.8)	88.4 (77.9‐94.5)	94.1 (84.9‐98.1)	81.6 (70.7‐89.2)
GPT-a	15.6 (5.9‐33.5)	0.0 (0.0‐17.2)	25.0 (12.1‐43.8)	87.0 (76.2‐93.5)	100.0 (93.3‐100.0)	84.2 (73.6‐91.2)
GPT-b	25.0 (12.1‐43.8)	0.0 (0.0‐17.2)	18.8 (7.9‐37.0)	89.9 (79.6‐95.5)	94.1 (84.9‐98.1)	84.2 (73.6‐91.2)
GPT-c	31.2 (16.7‐50.1)	0.0 (0.0‐17.2)	18.8 (7.9‐37.0)	91.3 (71.4‐96.4)	97.1 (88.8‐99.5)	82.9 (72.2‐90.2)

a Benign-malignant differentiation.

b DeepSeek: DeepSeek-R1.

c Basic patient information and imaging findings.

d Scenario A plus chief complaint or clinical history.

e Scenario B plus key laboratory results.

f Gemini: Gemini 2.5 Pro.

g GPT: GPT-4o.

h Disease diagnosis.

### BI-RADS Classification Consistency Assessment

Overall, diagnostic accuracy was highest for GPT-4o (range 60.2%‐64.8%), followed by DeepSeek-R1 (range 58.3%‐63.9%) and Gemini 2.5 Pro (range 50%‐55.6%; Table 3, Figure 2C and Figure 3C). A complete matrix of *P* values for all pairwise comparisons regarding BI-RADS classifications is provided in Table S3 in Multimedia Appendix 3.

## Discussion

### Principal Findings

While numerous studies have investigated the utility of LLMs for generating the impression section of radiology reports from images or findings [19-22], the diagnostic accuracy of these models across diverse clinical scenarios remains underexplored. This study evaluated the impact of varying clinical input information on the diagnostic accuracy of general-purpose LLMs (DeepSeek-R1, Gemini 2.5 Pro, and GPT-4o) and compared their performance with that of radiologists in real-world diagnostic tasks. While diagnostic accuracy generally improved with the addition of clinical information, this improvement was heterogeneous across models and disease types. In most cases, the addition of clinical history and laboratory results improved the diagnostic accuracy of the model (eg, in the liver cohort, the diagnostic accuracy in scenario C was higher than in scenario A for DeepSeek-R1 [77/101, 76.2%, vs 69/101, 68.3%; unadjusted *P*=.06, adjusted *P*>.99], Gemini 2.5 Pro [76/101, 75.2%, vs 69/101, 68.3%; unadjusted *P*=.10; adjusted *P*>.99], and GPT-4o [73/101, 72.3%, vs 65/101, 64.4%; unadjusted *P*=.06; adjusted *P*>.99]), and this result is consistent with those of Ueda et al [24]. However, we also observed exceptions: In the lung cohort, GPT-4o achieved its highest accuracy in scenario A (68/92, 73.9%). In the breast cohort, the diagnostic accuracy of DeepSeek-R1 peaked in scenario A (73/108, 67.6%) but decreased to 65.7% (71/108) with the addition of laboratory tests; there was no evidence of a difference between the scenarios (adjusted *P*>.99). This suggests that the addition of laboratory test results has less or even a negative impact on lung and breast diseases in diagnosis. This discrepancy may be because serum tumor markers such as alpha-fetoprotein, carbohydrate antigen 19‐9, and carcinoembryonic antigen play an essential role in the diagnosis of liver diseases [31]. It is also possible that the LLM may misinterpret the addition of extra information as “noise” rather than “signal,” thereby interfering with its diagnostic logic based on image findings and history. However, we emphasize that this is only a descriptive analogy, not a mechanistic claim, and the precise reasons for this performance degradation remain unclear.

### Comparison With Prior Work

Although providing comprehensive clinical information was associated with numerically higher diagnostic accuracy, the improvement was not statistically significant, and overall model performance remained inferior to that of radiologists. Zhang et al [20] reported similar results, noting that even their specialized model struggled to match radiologists’ performance in key dimensions, such as providing a “specific diagnosis” (647/1014, 63.8%). Interestingly, we observed that specific models achieved higher diagnostic accuracy in the breast cohort than radiologists. This discrepancy likely stems from the study’s rigorous evaluation criteria, which classified reports with unspecified case names as BI-RADS categories 0–4b as “inconsistent,” a rule that may have artificially underestimated the radiologists’ performance.

Kim et al [32] reported performance variations across radiologic subspecialties, a finding consistent with our results. Additionally, while the model demonstrated good diagnostic accuracy for common diseases, such as hepatocellular carcinoma and lung adenocarcinoma, a granular analysis of the “other” category revealed poor performance when handling rare and complex lesions. The model exhibited a tendency toward “shortcut learning,” a phenomenon frequently driven by confounding bias [33]. For example, Rueckel et al [34] demonstrated that an AI system designed to detect pneumothorax relied on the presence of thoracic drainage tubes instead of learning features intrinsic to the pathology itself. Similarly, in our study, when hepatic adenoma was misclassified as focal nodular hyperplasia (FNH), the model appeared to treat arterial phase hyperenhancement and persistent enhancement as sufficient criteria for FNH, thereby failing to incorporate a key discriminative feature where the central scar in FNH typically demonstrates delayed-phase hyperintensity. More broadly, the model appeared to rely heavily on combinations of demographic features and coarse imaging patterns, which may bias predictions toward population-level associations rather than individualized assessments. For instance, cases characterized as “young female with persistent enhancement” were frequently predicted as hepatic adenoma, whereas “young male without underlying liver disease but with a central scar” tended to be classified as FNH. A similar pattern was observed in pulmonary diagnoses, where a strong association between “middle-aged female” and lung adenocarcinoma appeared to underpin numerous misclassifications. Although lung adenocarcinoma is indeed more prevalent among women who have never smoked [35], the model’s reliance on statistical regularities may bias predictions toward more common diseases. For example, lung adenocarcinoma and sclerosing pneumocytoma share similar demographic profiles because both are more frequent in middle-aged women. In such cases, dependence on population-level patterns may lead the model to favor the more prevalent diagnosis, resulting in the underrecognition of rarer conditions such as sclerosing pneumocytoma. This limitation is particularly critical in clinical settings where the tolerance for diagnostic error is low [36]. We observed that GPT-4o achieved 0% accuracy for benign lung lesions across all 3 scenarios, indicating that every benign lesion in the lung cohort was classified as malignant. The clinical safety implications of this finding warrant particular attention. In a real-world clinical setting, such systematic overdiagnosis could lead to unnecessary invasive procedures, heightened patient anxiety, and substantial downstream health care costs. This failure mode underscores a broader deployment risk: current general-purpose LLMs may lack the conservative judgment required for low-tolerance clinical tasks, particularly when distinguishing benign from malignant conditions. In addition, automation bias in the model may lead to an imbalanced weighting of multimodal evidence. During diagnostic reasoning, the model may assign disproportionate importance to low-specificity imaging features, such as “possible biliary dilatation,” while failing to adequately integrate critical negative clinical findings and laboratory results. Consistent with our observations, Zack et al [37] reported that aggregate evaluation metrics can obscure underlying biases in individual cases, particularly those relating to demographic attributes.

We also observed performance variations among the models. Results indicated that model performance was consistent across input scenarios for the liver and breast cohorts. Conversely, performance for the lung cohort exhibited greater variability. We hypothesize that this discrepancy stems from differences in imaging modalities, as MRI is a multiparametric and multisequence modality that provides richer information than CT. Zheng et al [21] supported this observation and reported that the odds ratio of MRI versus CT was 3.46, with a 95% CI of 2.56 to 4.67. DeepSeek-R1 demonstrated the highest performance in assessing liver and breast diseases. This finding may be attributable to the use of Chinese-language inputs, given that DeepSeek-R1 was optimized for Chinese text during training, whereas Gemini 2.5 Pro and GPT-4o were not. Prior studies have reported substantial performance variability across languages in general-purpose LLMs. For example, Cozzi et al [38] demonstrated that human-LLM agreement for BI-RADS classification decreased markedly when moving from English to Italian and Dutch, while Meddeb et al [39] showed that translation quality varies significantly across different model-language pairs.

### Limitations

This study has several limitations.

First, a core limitation of this study is the inherent asymmetry in the comparison between LLMs and human radiologists. The experimental design comprised 3 progressively enriched, text-based clinical scenarios specifically constructed for LLM evaluation. In contrast, the historical radiologist impressions used as a comparator were generated in real-world clinical settings, where radiologists likely had access to comprehensive electronic health records at the time of diagnosis, including prior imaging studies, longitudinal clinical documentation, and laboratory data. This imbalance in information availability introduces a significant source of confounding and limits the validity of direct performance comparisons. This study design, while pragmatic for exploring LLM-based diagnostic reasoning, does not support a controlled or fully comparable assessment of radiologist-level performance. Future prospective studies should adopt a symmetrical evaluation framework, in which both LLMs and radiologists are provided with identical inputs under matched conditions. Such designs would enable a more rigorous and unbiased assessment of comparative diagnostic performance. For breast BI-RADS classification, the final radiologist impressions served as the reference standard. This design makes the model versus radiologist comparison structurally circular, as the radiologist defines the correct answer and necessarily achieves 100% accuracy. Model performance for this task should therefore be interpreted as agreement with the radiologist reference standard rather than as a direct comparison of diagnostic capability. Additionally, in the breast cohort, the strict classification rule used to define diagnostic consistency may have artificially lowered radiologist accuracy, thereby lowering the baseline for comparison with the models. As a result, the observed performance gap in this cohort may partly reflect the stringency of the evaluation framework rather than a true difference in diagnostic capability, representing an inherent comparability constraint. This limitation should therefore be considered when interpreting the comparative findings for the breast cohort.

Second, an inherent limitation of this study is that the models evaluated text-based descriptive findings rather than the medical images themselves. Consequently, the diagnostic performance ceiling of the models is entirely dependent on the visual perception and descriptive accuracy of the radiologists who originally authored the reports. If a subtle but diagnostically relevant imaging feature was omitted, understated, or ambiguously phrased in the text, the model necessarily lacked access to that information and could not incorporate it into its diagnostic reasoning. Future studies should develop multimodal model architectures that integrate imaging and text inputs to overcome the inherent constraints of single-modality text-based evaluation.

Third, a limitation concerns the asymmetry in clinical information provided across disease cohorts. In scenario B, breast cases were accompanied by relatively comprehensive clinical histories, including medical, menstrual, and family information. In contrast, liver and lung cases were provided with only the chief complaint. This asymmetry was not due to an inability to retrieve available clinical history, but rather to reflect the absence of equivalently structured data for the liver and lung cohorts in the retrospective data source. This imbalance in contextual information introduces a potential source of confounding and limits the validity of direct cross-organ performance comparisons. While this design partially reflects real-world variability in clinical documentation across organ systems, it nonetheless constitutes a structural inconsistency within the experimental framework. Future prospective studies should adopt standardized clinical information templates across all disease types, ensuring that models are evaluated under matched informational conditions to enable more rigorous and unbiased cross-organ comparisons. Additionally, several aspects of the data input design may have elevated input quality relative to routine clinical practice, thereby limiting external validity. First, in scenario C, we provided only key laboratory markers relevant to each disease type and manually removed the vast majority of laboratory data typically found in real-world electronic health records, effectively and artificially “sanitizing” the dataset. Second, these laboratory values were supplied without accompanying institutional normal reference ranges; because reference intervals vary by assay and equipment, the models may have been forced to rely on generalized reference intervals from pretraining data, potentially introducing uncertainty into the interpretation of laboratory values and contributing to diagnostic error. Furthermore, the exclusion of incomplete or ambiguous radiology reports, for example, 317 cases in the lung cohort, further elevated input quality relative to routine clinical practice. Future studies should use complete, unfiltered laboratory datasets with explicit institutional reference ranges to more accurately assess model robustness under clinically realistic conditions.

Fourth, language represents an unavoidable source of potential bias. As the clinical data were provided in Chinese and different models may exhibit varying degrees of native multilingual optimization, the observed performance differences may be partially driven by differences in linguistic comprehension rather than solely reflecting variations in clinical reasoning ability. Future studies incorporating multilingual inputs, as well as models with different primary language optimization strategies, are warranted to more rigorously disentangle linguistic effects from true diagnostic reasoning performance.

Fifth, the study cohort included only surgically resected cases with histopathological confirmation, which introduces a substantial selection bias. In routine clinical practice, many typical benign lesions (eg, hepatic hemangiomas or breast fibroadenomas) are confidently diagnosed based on imaging alone and therefore do not undergo surgical resection. Consequently, the benign cases in this study are likely enriched for atypical or diagnostically challenging lesions, which may partly explain the relatively low diagnostic accuracy observed for benign diseases. In addition, the statistical power for analyses of benign subgroups was limited, as reflected by wide CIs and unstable estimates. These findings should therefore be interpreted with caution, and the diagnostic performance of the models for benign conditions requires further validation in larger and more representative cohorts.

Sixth, no prospective sample size calculation was performed prior to data collection. According to the STARD (Standards for Reporting of Diagnostic Accuracy Studies) 2015 guidelines (item 14), a priori sample size estimation is recommended to ensure adequate precision and reliability of diagnostic accuracy studies. The absence of such a formal calculation in this study may have affected the precision of the reported performance estimates. Future prospective studies should incorporate predefined sample size calculations to ensure sufficient statistical power and more precise estimation of diagnostic accuracy.

Seventh, it should be noted that the evaluation in this study was based on a strict binary rubric that credited only the definitive final diagnosis. The models were prompted to provide both a final diagnosis and a differential diagnosis; however, the differential diagnosis sections were typically very broad and nondiscriminatory. Crediting a case as correct based solely on the presence of the correct diagnosis anywhere in the output would have artificially inflated the apparent performance. We therefore adopted a conservative approach, while acknowledging that the strict rubric may underestimate the models’ capacity for nuanced clinical reasoning when the correct diagnosis is appropriately prioritized.

Eighth, all clinical text was manually deidentified prior to input, with patient names, medical record numbers, and institutional identifiers removed. However, certain dates embedded in the clinical history, such as the last menstrual period date and other dated clinical events, were not uniformly removed or generalized during the initial data preparation. This represents a limitation in our deidentification protocol and should be acknowledged as a privacy consideration. Future studies using public LLM interfaces should adopt more rigorous deidentification procedures, including the removal or generalization of all date information, to fully comply with best practices for patient data protection.

Finally, the study relied on single-shot prompting, which does not fully capture the probabilistic nature and inherent stochasticity of general-purpose LLMs. As a result, the stability, reproducibility, and intramodel variability of diagnostic outputs could not be systematically evaluated, and the potential variation in reasoning across repeated inference runs remains unquantified. In addition, the prompt templates used in this study were relatively basic and have not undergone extensive optimization. As such, the potential benefits of more advanced prompt engineering strategies were not fully explored in this foundational evaluation. To more rigorously assess diagnostic robustness, future studies should adopt multi-iteration inference protocols. Such designs would enable estimation of output variance, systematic evaluation of hallucination rates, and construction of CIs for model-level performance. Coupling repeated inference with refined prompt optimization strategies will be essential for more comprehensively characterizing the intrinsic reliability of these models in clinical applications.

### Conclusions

In conclusion, we evaluated the diagnostic accuracy of DeepSeek-R1, Gemini 2.5 Pro, and GPT-4o in liver, lung, and breast diseases across progressive information input scenarios and compared their accuracy with that of radiologists. A numerical trend toward improvement was observed when systematically increasing the dimensionality of clinical information, but no statistically significant difference was demonstrated. Previous studies have demonstrated the potential of LLMs to automatically extract clinical histories [40,41]. Future clinical AI systems may leverage LLMs to integrate imaging, clinical history, and laboratory data, thereby further improving diagnostic accuracy.

## Supplementary material

10.2196/94904 Multimedia Appendix 1 Template examples for different diseases.

10.2196/94904 Multimedia Appendix 2 Model parameters and radiologist characteristics.

10.2196/94904 Multimedia Appendix 3 All statistical values in the study.

## Abbreviations

BI-RADS: Breast Imaging Reporting and Data System
CT: computed tomography
FNH: focal nodular hyperplasia
LLM: large language model
MRI: magnetic resonance imaging
STARD: Standards for Reporting of Diagnostic Accuracy Studies
