# Supplementary material for: Incremental Diagnostic Value of Clinical Information for Large Language Models Across Multiple Organs: Retrospective Study
Source: J Med Internet Res. 2026 Aug 26;28:e94904. doi: 10.2196/94904 (PMC13519069; doi:10.2196/94904)
Supplement: Supplementary file 1 [file jmir-v28-e94904-s001.docx]

**Multimedia Appendix 1:** Template Examples for Different Diseases

## **Table S1:** Clinical Information Input Templates for Large Language Models (model inputs were in Chinese, with English translations for clarity).

1. **Liver Template**

① Basic Information: Gender, Age

② Imaging Findings:

③ Chief Complaint:

④ Clinical Data:

Prothrombin Time (PT) = __ (S)

International Normalized Ratio (INR) = __

Hepatitis B Surface Antigen (HBsAg) = __ (IU/mL)

Hepatitis B Surface Antibody (HBsAb) = __ (mIU/mL)

Hepatitis B e Antigen (HBeAg) = __ (S/CO)

Hepatitis B e Antibody (HBeAb) = __ (S/CO)

Hepatitis B Core Antibody (HBcAb) = __ (S/CO)

Hepatitis C Virus Antibody (Anti-HCV) = __ (S/CO)

Alanine Aminotransferase (ALT) = __ (U/L)

Aspartate Aminotransferase (AST) = __ (U/L)

Lactate Dehydrogenase (LDH) = __ (U/L)

Gamma-Glutamyl Transferase (GGT) = __ (U/L)

Alkaline Phosphatase (ALP) = __ (U/L)

Total Bilirubin (TBIL) = __ (μmol/L)

Albumin (ALB) = __ (g/L)

Carbohydrate Antigen 19-9 (CA19-9) = __ (U/ml)

Alpha-Fetoprotein (AFP) = __ (ng/ml)

Carcinoembryonic Antigen (CEA) = __ (ng/ml)

Please provide the final diagnosis and differential diagnosis based on all the information provided above**.**

2. **Lung Template**

① Basic Information: Gender, Age

② Imaging Findings:

③ Chief Complaint:

④ Clinical Data:

C-Reactive Protein (CRP) = __ (mg/dl)

Absolute Neutrophil Count = __ (×10^9/L)

Absolute Lymphocyte Count = __ (×10^9/L)

Absolute Monocyte Count = __ (×10^9/L)

Hemoglobin (HGB) = __ (g/L)

Platelet Count (PLT) = __ (×10^9/L)

Carbohydrate Antigen 125 (CA125) = __ (U/ml)

Cytokeratin 19 Fragment (CYFRA 21-1) = __ (ng/ml)

Neuron-Specific Enolase (NSE) = __ (ng/ml)

Squamous Cell Carcinoma Antigen (SCC) = __ (ng/ml)

Carcinoembryonic Antigen (CEA) = __ (ng/ml)

Pro-Gastrin-Releasing Peptide (ProGRP) = __ (pg/ml)

Please provide the final diagnosis and differential diagnosis based on all the information provided above.

3. **Breast Template**

① Basic Information: Gender, Age

② Imaging Findings:

③ Chief Complaint + Medical History: (Past Medical History, Menstrual History, Marital and Childbearing History, Family History)

④ Clinical Data:

Carbohydrate Antigen 125 (CA125) = __ (U/ml)

Carbohydrate Antigen 15-3 (CA15-3) = __ (U/ml)

Carcinoembryonic Antigen (CEA) = __ (ng/ml)

Please provide the final diagnosis (Disease Name + BI-RADS Score) and differential diagnosis based on all the information provided above.

## **Table S2:** Examples of Clinical Information Input for Large Language Models (Model input data are in Chinese, with English translations provided for ease of understanding). Figure and image references appearing in the input templates reflect the original radiologists' notations for specific image series or slices and were retained as part of the unaltered report text.

**1. Liver Disease (DeepSeek-R1 )**

**Input:**

① Basic Information: Male, 65 years old

② Imaging Findings: Cirrhosis; an abnormally signal-intensity mass is noted in the right lobe of the liver, measuring approximately 6.1 × 5.7 cm (Fig. 4-7). On T1-weighted images (T1WI), the mass exhibits heterogeneous signal intensity with patchy areas of slightly lower signal intensity; no signal attenuation is observed on T1WI in the inverted phase. On T2-weighted images (T2WI)/FS, the mass shows heterogeneous, slightly increased signal intensity; high signal on DWI; marked enhancement during the arterial phase of contrast-enhanced imaging, with reduced enhancement during the portal and delayed phases, lower than that of normal liver parenchyma; multiple nodules surrounding the mass, the largest measuring approximately 1.4 × 0.8 cm (Fig. 4-7), showing slightly low signal on T1-weighted images, slightly high signal on T2-weighted/FS images, and high signal on DWI; marked enhancement during the arterial phase of contrast-enhanced imaging, with reduced enhancement during the portal and delayed phases. Abnormal signal-intensity nodule in the right kidney, approximately 1.0 × 0.7 cm in size (Fig. 33-53); T1WI: high signal intensity; T2WI/FS: high signal intensity; no enhancement observed on contrast-enhanced imaging. Abnormal signal-intensity lesion in the left kidney, the largest of which is approximately 0.3 cm (Fig. 2-17); T2WI/FS: high signal intensity; no enhancement observed on contrast-enhanced imaging. No definite abnormalities were observed in the gallbladder, pancreas, spleen, or bilateral adrenal glands. No clearly enlarged lymph nodes were found in the abdominal cavity or retroperitoneum. No ascites was observed.

③ Chief complaint: Discovery of a liver mass more than one week ago; Physical examination: Discovery of a liver mass more than one week ago; Denies history of hepatitis

④ Clinical Data: Prothrombin time (PT) = 11.1 (S), International Normalized Ratio (INR) = 0.97, Hepatitis B surface antigen (HBsAg) = 0 (IU/mL), Hepatitis B surface antibody (HBsAb) = 3.44 (mIU/mL), Hepatitis B e antigen (HBeAg) = 0.359 (S/CO), Hepatitis B e-antibody (HBeAb) = 1.14 (S/CO), Hepatitis B core antibody (HBcAb) = 5.41 (S/CO), Anti-HCV = 0.11 (S/CO), Alanine aminotransferase (ALT) = 41 (U/L), Aspartate aminotransferase (AST) = 30.1 (U/L), Lactate dehydrogenase (LDH) = 191.3 (U/L), γ-glutamyltransferase (GGT) = 101.5 (U/L), alkaline phosphatase (ALP) = 78.2 (U/L), total bilirubin (TBIL) = 12.8 (μmol/L), albumin (ALB) = 42.5 (g/L), CA19-9 = 7.73 (U/mL), Alpha-fetoprotein (AFP) = 4.1 (ng/mL), Carcinoembryonic Antigen (CEA) = 2.05 (ng/mL).

Please provide a final diagnosis and differential diagnosis based on all the information provided above.

**Output：**

Based on the complete information you provided, the following diagnosis and differential diagnosis analysis is presented:

Final Diagnosis

1. Mass in the right lobe of the liver (6.1 × 5.7 cm) and multiple surrounding nodules:

Highly consistent with hepatocellular carcinoma (HCC). Imaging findings: Typical “rapid-on, rapid-off” enhancement pattern: marked enhancement in the arterial phase, with enhancement attenuation in the portal phase and delayed phase (lower than the hepatic parenchyma).

Diffusion restriction: High signal on DWI suggests cellular density.

Cirrhosis background: The patient has cirrhosis, which is a high-risk factor for HCC.

Satellite lesions (multiple peripheral nodules): The enhancement pattern is consistent with the primary lesion, consistent with intrahepatic metastasis of HCC.

Other features: Slightly heterogeneous hyperintensity on T2WI, isointense signal on T1WI with patchy hypointensity (possibly necrosis/hemorrhage) .

2. Right renal nodule (1.0 × 0.7 cm) and left renal microlesion (0.3 cm):

Consistent with benign cysts: High signal on T1WI and T2WI (consistent with high protein content in cyst fluid or hemorrhagic cysts); no enhancement on contrast-enhanced imaging.

3. Cirrhosis (cause undetermined):

No evidence of viral hepatitis: HBsAg negative, Anti-HCV negative.

Other causes should be considered: alcoholic, metabolic (e.g., fatty liver), occult hepatitis B (HBcAb positive but HBsAg negative), or autoimmune liver disease, etc.

Differential Diagnosis

Differential diagnosis of hepatic masses: Intrahepatic Cholangiocarcinoma (ICC): Findings not supportive: Typical enhancement pattern for ICC: marginal enhancement in the arterial phase, followed by progressive centripetal enhancement in the delayed phase (this case shows “rapid inflow and outflow”). Often accompanied by elevated CA19-9 (normal in this case). Rarely occurs on a background of cirrhosis. Metastatic liver cancer: Incompatible findings: No history of primary tumor (e.g., colorectal cancer, gastric cancer, etc.). Metastases typically present with a “bull’s-eye sign” or annular enhancement; “fast-in, fast-out” is rare.

Tumor markers (CEA, CA19-9, AFP) are all normal. Hepatic hemangioma: Consistent findings: Typically presents with marked hyperintensity on T2-weighted images (“lightbulb sign”) and “slow-in, slow-out” enhancement on contrast-enhanced scans (this case shows “fast-in, fast-out”). DWI typically shows no high signal (high signal on DWI in this case suggests malignancy). Focal nodular hyperplasia (FNH): Inconsistent finding: Marked enhancement in the arterial phase of contrast-enhanced imaging, but isochromatic or slightly hyperintense in the portal and delayed phases (in this case, enhancement is diminished). Central scarring appears as a hyperintense signal on T2WI with delayed enhancement (this case lacks this feature). Hepatic adenoma: Inconsistent findings: Commonly occurs in young women (associated with oral contraceptives); this case involves an elderly male. T1-weighted images often show reduced signal intensity in the inversion phase (suggesting lipid content); this case lacks reduced signal intensity in the inversion phase. Differential diagnosis of renal nodules: Renal cell carcinoma (e.g., papillary renal cell carcinoma): Inconsistent findings: Contrast-enhanced scans typically show enhancement (this case shows no enhancement). T1-weighted images typically show low signal intensity (the high signal intensity in this case is consistent with a cyst). Complex cyst/infectious cyst:

Imaging findings are consistent with a benign cyst (high T2 signal intensity, no enhancement); the high T1 signal intensity in the right renal nodule may represent hemorrhage or high protein content.

Key clinical implications. Reliability of HCC diagnosis: LI-RADS Category 5 (highly suggestive of HCC): Based on a background of cirrhosis + typical imaging features (enhancement in the arterial phase + washout in the portal phase + restricted DWI). Normal AFP does not rule out HCC: Approximately 30% of HCC patients have normal AFP levels (AFP = 4.1 ng/ml in this case).

Recommendations for further management: Liver lesions: Multidisciplinary consultation (hepatobiliary surgery, interventional radiology, oncology); surgical resection or local ablation is the first-line treatment (if applicable). If surgery is not feasible, consider TACE (transarterial chemoembolization) or targeted/immunotherapy. Complete screening for the causes of cirrhosis (e.g., iron metabolism, antibodies for autoimmune liver disease, assessment of alcoholic liver disease).

Renal lesions: The right renal cyst requires follow-up (MRI follow-up in 1 year); the left renal microcyst is clinically insignificant.

Summary. Final diagnosis: Hepatocellular carcinoma (HCC) with intrahepatic metastasis (on a background of cirrhosis, LI-RADS Category 5).

Hemorrhagic/proteinaceous cyst in the right kidney; microcyst in the left kidney. Cirrhosis (cause to be determined). Immediate management of the hepatic mass is required, along with thorough investigation of the cause of cirrhosis to guide long-term management.

**2. Lung Disease (Gemini 2.5 Pro )**

**Input:**

① Basic Information: Female, 32 years old

② Imaging Findings: Figure 4-27 shows a nodule in the right lower lobe of the lung with clear borders, measuring approximately 1.8 × 1.6 × 1.8 cm. The mean CT value on non-contrast imaging is approximately 28 HU, and the mean CT value after contrast enhancement is approximately 69 HU. Coarse, tortuous vessels are visible within the nodule.

No clearly enlarged lymph nodes were observed in the mediastinum or at both hilum.

No effusion was observed in the pleural cavity or pericardium.

③ Chief Complaint: Follow-up for pulmonary nodule; Physical Examination: Follow-up for pulmonary nodule

④ Clinical Data: C-reactive protein (CRP) = 0.11 (mg/dL), absolute neutrophil count = 4.12 (×10^9/L), absolute lymphocyte count = 2.82 (×10^9/L), absolute monocyte count = 0.28 (×10^9/L), Hemoglobin HGB = 146 (g/L), Platelet Count PLT = 377 (×10^9/L), Carbohydrate Antigen 125 CA125 = 10.7 (U/ml), Cytokeratin 19 Fragment cyfra21-1 = 1.13 (ng/mL), Neuron-Specific Enolase (NSE) = 10.9 (ng/mL), squamous cell carcinoma-associated antigen (SCC) = 1.54 (ng/mL), carcinoembryonic antigen (CEA) = 0.51 (ng/mL), pro-gastrin-releasing peptide (ProGRP) = 22.6 (pg/mL)

Please provide a final diagnosis and differential diagnosis based on all the information provided above.

**Ouput:**

Summary of Key Findings Patient: 32-year-old female, young patient. Chief complaint: Follow-up evaluation of a pulmonary nodule, indicating that the lesion persists. Key imaging features: Location: Right lower lobe; solitary. Morphology: A solid nodule with well-defined borders. Size: Approximately 1.8 × 1.6 × 1.8 cm; classified as a medium-sized nodule. CT Values and Enhancement: Non-contrast CT value: 28 HU (soft tissue density). Post-contrast CT value: 69 HU; enhancement ΔCT = 41 HU. This represents very significant enhancement (typically, an increase of >15–20 HU is considered significant), suggesting a highly vascularized lesion. Key Findings: “Enlarged, tortuous vessels visible within the lesion”—this is the most characteristic radiological feature of this case, commonly referred to as the “vascular embedding sign” or “feeding artery sign,” and is highly suggestive of the diagnosis. Lymph Nodes and Pleural Space: No mediastinal lymphadenopathy and no pleural effusion, suggesting no obvious signs of metastasis. Key laboratory findings: Inflammatory markers (CRP, neutrophils): All within normal ranges, essentially ruling out active infection. Tumor markers (CA125, Cyfra 21-1, NSE, SCC, CEA, ProGRP): All tested lung cancer-related tumor markers were negative, with very low levels. This significantly reduces the likelihood of common types of lung cancer (adenocarcinoma, squamous cell carcinoma, small cell carcinoma). Final diagnosis (most likely diagnosis): Sclerosing pneumocytoma (formerly known as sclerosing hemangioma). Basis for Diagnosis: 1. High epidemiological consistency: Sclerosing pneumocytoma is a low-grade or borderline malignant tumor that commonly occurs in young and middle-aged women; the patient’s profile as a 32-year-old woman is highly typical. 2. High consistency with imaging features: It typically presents as a solitary, well-defined round or oval nodule, consistent with this case. Significant progressive enhancement is a characteristic feature. In this case, the enhancement value of 41 HU indicates a rich blood supply, consistent with the pathological structure of sclerosing pulmonary cell carcinoma (vascular-rich papillary and sclerotic areas). “Enlarged, tortuous vessels visible within the lesion,” also known as the “vessel penetrating sign,” is a highly characteristic feature of sclerosing pulmonary cell carcinoma and provides strong diagnostic support. 3. Laboratory findings: This tumor typically does not produce conventional lung cancer tumor markers; therefore, a negative result for the full panel of serum tumor markers perfectly supports this diagnosis and serves as an important basis for ruling out common malignant tumors. 4. Clinical presentation: The patient had no specific symptoms and was only found during a follow-up examination after an initial physical examination, consistent with the tumor’s typically slow growth and lack of obvious clinical symptoms. Differential Diagnosis: Although pulmonary sclerosing cell tumor is the most likely diagnosis, the following possibilities should still be considered prior to pathological confirmation: 1. Pulmonary Hamartoma Supporting evidence: The most common benign lung tumor, occurring at any age, and typically having well-defined borders. Points against/differences: Classic hamartomas contain fatty density (-120 HU to -40 HU) or “popcorn-like” calcifications, which are not mentioned in this case. Although hamartomas without fat or calcifications do exist, and some may show mild to moderate enhancement, the presence of “internally thickened, tortuous vessels”—a hallmark of significant blood supply—is relatively rare. 2. Carcinoid Tumor Supporting evidence: A neuroendocrine tumor that can occur in younger patients. It typically presents as a well-defined nodule with an extremely rich blood supply, resulting in very marked enhancement. Non-supporting evidence/differences: Although typical carcinoids are common, as a neuroendocrine tumor, the marker NSE is sometimes elevated. In this case, the NSE level is normal (10.9 ng/ml), which somewhat reduces the likelihood of a carcinoid tumor, but cannot completely rule it out (especially since NSE is not highly sensitive for typical carcinoids). 3. Well-differentiated Adenocarcinoma Supporting Evidence: Lung adenocarcinoma is the most common type of lung cancer in women and non-smokers and can present as a solitary nodule. Contradictory Findings/Differences: Age: Lung cancer is relatively rare in a 32-year-old. Tumor Markers: Markers such as CEA and CA125 are often elevated in patients with lung adenocarcinoma; in this case, all were negative and the levels were extremely low, which argues against adenocarcinoma. Imaging Features: Although adenocarcinoma is also well-vascularized, the typical appearance of “enlarged, tortuous vessels within the lesion” is less characteristic than that seen in sclerosing pulmonary cell carcinoma or carcinoid tumors. 4. Arteriovenous Malformation (AVM) Supporting Evidence: By definition, an AVM is an abnormal cluster of blood vessels; therefore, “enlarged, tortuous vessels” are a core feature and exhibit extreme enhancement. Points against/Differences: AVMs typically show one or more clearly identifiable feeding arteries and draining veins, and the lesion consists primarily of a vascular mass. In contrast, this case describes “vessels within the nodule,” suggesting the lesion contains a solid soft tissue component (CT value 28 HU), which is more consistent with a tumor rather than a simple vascular malformation. 5. Inflammatory Pseudotumor Supporting Evidence: May present as a well-defined, solitary mass; some cases may also show marked enhancement. Non-supporting Evidence/Differences: The patient’s CRP and white blood cell count are normal, with no laboratory findings supporting infection or inflammation; this is therefore a low probability. Conclusion and Recommendations: Based on all available information, sclerosing pulmonary cell tumor is the primary diagnosis in this case. This is a case with highly specific imaging and laboratory findings. However, imaging diagnosis cannot replace a pathological diagnosis. For a solid nodule with a diameter approaching 2 cm and a rich blood supply, definitive diagnosis requires histological evidence. Recommendation: Clinical Decision: Given that the nodule is not small (>8 mm) and is solid and well-vascularized, surgical resection is indicated regardless of whether the final diagnosis is sclerosing pulmonary cell tumor (low malignant potential) or carcinoid (low malignant potential). Next Steps: Thoracoscopic wedge resection is recommended. This serves both as the definitive diagnostic method (intraoperative frozen section analysis + postoperative paraffin-embedded histology) and as a curative treatment. For a nodule of this size and location, minimally invasive surgery is the safe and effective standard of care.

**3. Breast Disease (GPT-4o )**

**Input:**

① Basic Information: Female, 49 years old

② Imaging Findings: Bilateral breast tissue appears dense, with moderate enhancement of the background.

An irregular mass is visible in the upper inner quadrant of the right breast, measuring approximately 1.7 × 1.5 × 1.4 cm (Fig. 29-28/35-53). The margins are slightly irregular; the lesion appears as slightly hyperintense on T2WI/water, hyperintense on DWI, and shows marked heterogeneous enhancement on contrast-enhanced imaging.

Scattered punctate enhancing lesions are present in both breasts, with the largest measuring approximately 0.3 cm (Fig. 33-54); these show isointense signal on T2WI/water and DWI, with progressive enhancement on contrast-enhanced imaging.

No thickening of the skin over the breasts is observed, and the nipples are not inverted.

Multiple lymph nodes are noted in the right axilla within the scan field, with the largest measuring approximately 0.6 cm in short axis diameter (Fig. 33-32). No clearly enlarged lymph nodes are observed in the left axilla.

③ Chief complaint: A nodule in the upper quadrant of the right breast, approximately 1 cm in size. External hospital breast ultrasound findings: A nodule at the 12 o’clock position in the right breast, BIRADS 4B, approximately 2.1 cm in size. Physical examination: Post-lung cancer surgery

Medical history: In 2021, a Mirena IUD was inserted due to endometrial thickening. In November 2022, a left lobectomy was performed due to a space-occupying lesion in the lung; postoperative pathology revealed early-stage lung cancer, and no specific postoperative treatment was administered. Denies history of infectious diseases such as tuberculosis or hepatitis. Denies history of hypertension, heart disease, or diabetes. Denies history of drug or food allergies. Denies history of trauma, fat injections, or breast augmentation. Vaccination history unknown.

Menstrual history: Menarche at age 16, cycle length 7 days/30 days, last menstrual period on November 15, 2024. Currently in the premenopausal stage.

Marital and Reproductive History: Married at age 24; 3 pregnancies, 2 live births; breastfed; has one son and one daughter; family members are in good health.

Family History: No history of breast cancer or ovarian cancer; no other family history of cancer; father has a history of esophageal cancer.

④ Clinical Data: CA125 = 15.8 (U/ml), CA15-3 = 9.52 (U/ml), CEA = 1.37 (ng/ml)

Based on all the information provided above, please provide a final diagnosis (disease name + BI-RADS score) and differential diagnosis.

**Ouput:**

Based on the comprehensive medical history, imaging findings, and laboratory data you provided, the analysis is summarized as follows: ✅ Final Diagnosis: - Disease Name: Right breast cancer (suspected invasive breast cancer) - BI-RADS Score: Category 5 (BI-RADS 5) —— Strongly suggestive of malignancy (>95% probability of cancer). 🧠 Basis for Analysis: 🔬 Imaging findings suggestive of malignancy: - Mass morphology: A mass in the upper inner quadrant of the right breast, irregular in shape with slightly irregular margins, suggesting a non-benign lesion. - Signal characteristics: - T2-weighted imaging (T2WI): Slightly hyperintense signal (moderately common in malignant lesions) - Diffusion-weighted imaging (DWI): Hyperintense signal → Indicates restricted water diffusion, commonly seen in malignant tumors with high cellular density. - Contrast-enhanced imaging: Markedly heterogeneous enhancement → A typical presentation of malignant tumors. - Lymph nodes: Multiple lymph nodes in the right axilla, with the largest measuring 0.6 cm in short axis diameter; risk of regional metastasis. 🧪 Serum Tumor Markers: - CA125, CA15-3, and CEA are all within normal ranges → Early-stage breast cancer cannot be ruled out, as the sensitivity of these markers is limited. 🧑‍⚕️ Clinical Background: - Physical examination reveals a breast mass of approximately 2.1 cm; ultrasound at another hospital classified as BI-RADS 4B (moderate risk of malignancy) - History of early-stage lung cancer; vigilance is warranted for multiple primary tumors or metastases. 🩺 Differential Diagnosis: 1. Ductal Carcinoma In Situ (DCIS) - Typically presents with linear or branching calcifications, mild enhancement on MRI, and rarely forms large masses; - In this case, the mass is already large and shows heterogeneous enhancement, which is inconsistent with the characteristics of DCIS. 2. Benign breast tumors (e.g., fibroadenoma) - Benign lesions typically have well-defined borders, regular shapes, and homogeneous enhancement; - In this case, the mass has an irregular, irregular shape, a rough surface, and heterogeneous enhancement, which does not align with benign features. 3. Inflammatory lesions such as breast tuberculosis or granulomatous mastitis - Often accompanied by pain, skin redness and swelling, or fistula formation; - MRI typically shows annular enhancement, and DWI is often inconsistent; - This case shows no signs of inflammation, and the enhancement and signal patterns are consistent with a malignant tumor. 4. Breast metastases from lung cancer - Rare; typically multiple, bilateral, and well-defined; - This case presents as a solitary mass with typical malignant features on MRI, suggesting primary breast cancer. 🔍 Recommended next steps: - Breast fine-needle aspiration or core needle biopsy for definitive pathological diagnosis; - Supplementary mammography to assess calcification distribution; - Whole-body imaging to rule out distant metastases (especially given the history of lung cancer); - Multidisciplinary team (MDT) discussion to evaluate treatment options (surgery ± chemotherapy ± radiation therapy ± hormone therapy, etc.). If needed, I can help draft a clinical consultation recommendation or a breast cancer pathology analysis template. Is this required?
