# Supplementary material for: Incremental Diagnostic Value of Clinical Information for Large Language Models Across Multiple Organs: Retrospective Study
Source: J Med Internet Res. 2026 Aug 26;28:e94904. doi: 10.2196/94904 (PMC13519069; doi:10.2196/94904)
Supplement: Supplementary file 2 [file jmir-v28-e94904-s002.docx]

# **Multimedia Appendix 2:** Model parameters and radiologist characteristics

## **Table S1：**Radiologist characteristics.

| Characteristic | Liver(n=101) | Lung(n=92) | Breast(n=108) |
| --- | --- | --- | --- |
| Years of experience, mean (range) | 23(13-36) | 21(13-37) | 19(9-25) |
| Professional title |  |  |  |
| Attending physician | 7(6.9%) | 13(14.1%) | 58(53.7%) |
| Associate chief physician | 84(83.2%) | 57(62.0%) | 47(43.5%) |
| Chief physician | 10(9.9%) | 22(23.9%) | 3(2.8%) |

## **Table S2：**Model generation hyperparameters.

| Parameter | DeepSeek-R1 | Gemini 2.5 Pro | GPT-4o |
| --- | --- | --- | --- |
| Temperature | 0.6 | 1.0 | 0.7 |
| Top-p | 0.95 | 0.95 | 1.0 |
| Frequency penalty | — | — | 0 |

Note: All models were queried via their respective public consumer web interfaces. The parameter values listed are the documented defaults as specified in the official Application Programming Interface (API) documentation or technical reports for each model at the time of access; they were not independently set or verified in the web interface and are provided solely for reference.
