# Supplementary material for: Incremental Diagnostic Value of Clinical Information for Large Language Models Across Multiple Organs: Retrospective Study
Source: J Med Internet Res. 2026 Aug 26;28:e94904. doi: 10.2196/94904 (PMC13519069; doi:10.2196/94904)
Supplement: Supplementary file 3 [file jmir-v28-e94904-s003.docx]

# **Multimedia Appendix 3:** All statistical values in the study

## **Table S1：**Inter-rater reliability between the two primary raters, the Kappa coefficient, and its 95% confidence interval

| **Category** | **Cohen κ coefficient (95% CI)** |
| --- | --- |
| Benign-Malignant Differentiation | 0.978 (0.969, 0.988) |
| Disease Diagnosis | 0.980 (0.972, 0.988) |
| BI-RADS | 0.985 (0.974, 0.996) |
| All | 0.980 (0.976, 0.986) |

## **Table S2：**Summary of Diagnostic Performance Metrics. DeepSeek = DeepSeek-R1, Gemini = Gemini 2.5 Pro, GPT = GPT-4o.

| **Liver_benign-malignant differentiation** | | | | | |
| --- | --- | --- | --- | --- | --- |
|  | Accuracy | Sensitivity | Specificity | Pos Pred Value | Neg Pred Value |
| Radiologist | 0.901 | 0.986 | 0.719 | 0.883 | 0.958 |
| DeepSeek_a | 0.842 | 0.971 | 0.562 | 0.827 | 0.9 |
| DeepSeek_b | 0.881 | 0.986 | 0.656 | 0.861 | 0.955 |
| DeepSeek_c | 0.881 | 0.986 | 0.656 | 0.861 | 0.955 |
| Gemini_a | 0.832 | 1 | 0.469 | 0.802 | 1 |
| Gemini_b | 0.812 | 0.986 | 0.438 | 0.791 | 0.933 |
| Gemini_c | 0.842 | 0.986 | 0.531 | 0.819 | 0.944 |
| GPT_a | 0.772 | 1 | 0.281 | 0.75 | 1 |
| GPT_b | 0.812 | 1 | 0.406 | 0.784 | 1 |
| GPT_c | 0.851 | 0.986 | 0.562 | 0.829 | 0.947 |
| **Lung_benign-malignant differentiation** | | | | | |
|  | Accuracy | Sensitivity | Specificity | Pos Pred Value | Neg Pred Value |
| Radiologist | 0.87 | 1 | 0.5 | 0.85 | 1 |
| DeepSeek_a | 0.728 | 0.912 | 0.208 | 0.765 | 0.455 |
| DeepSeek_b | 0.772 | 0.956 | 0.25 | 0.783 | 0.667 |
| DeepSeek_c | 0.75 | 0.882 | 0.375 | 0.8 | 0.529 |
| Gemini_a | 0.761 | 1 | 0.083 | 0.756 | 1 |
| Gemini_b | 0.793 | 1 | 0.208 | 0.782 | 1 |
| Gemini_c | 0.717 | 0.956 | 0.042 | 0.739 | 0.25 |
| GPT_a | 0.75 | 1 | 0.042 | 0.747 | 1 |
| GPT_b | 0.728 | 0.941 | 0.125 | 0.753 | 0.429 |
| GPT_c | 0.728 | 0.971 | 0.042 | 0.742 | 0.333 |
| **Breast_benign-malignant differentiation** | | | | | |
|  | Accuracy | Sensitivity | Specificity | Pos Pred Value | Neg Pred Value |
| Radiologist | 0.917 | 0.934 | 0.875 | 0.947 | 0.848 |
| DeepSeek_a | 0.787 | 0.961 | 0.375 | 0.785 | 0.8 |
| DeepSeek_b | 0.806 | 0.974 | 0.406 | 0.796 | 0.867 |
| DeepSeek_c | 0.787 | 0.974 | 0.344 | 0.779 | 0.846 |
| Gemini_a | 0.778 | 0.987 | 0.281 | 0.765 | 0.9 |
| Gemini_b | 0.778 | 0.987 | 0.281 | 0.765 | 0.9 |
| Gemini_c | 0.778 | 0.987 | 0.281 | 0.765 | 0.9 |
| GPT_a | 0.759 | 0.974 | 0.25 | 0.755 | 0.8 |
| GPT_b | 0.75 | 0.974 | 0.219 | 0.747 | 0.778 |
| GPT_c | 0.75 | 0.961 | 0.25 | 0.753 | 0.727 |

## **Table S3：***P* Value Matrices for All Pairwise Comparisons using McNemar Tests across All Combinations of Models and Scenarios, **P* < .05, ***P* < .01, ****P* < .001. DeepSeek = DeepSeek-R1, Gemini = Gemini 2.5 Pro, GPT = GPT-4o. NA: Not applicable. For breast BI-RADS classification, the radiologist's assessment served as the reference standard; therefore, statistical comparison between the models and this reference standard is not defined.

| **Liver_benign-malignant differentiation** | | | | |
| --- | --- | --- | --- | --- |
| Model_1 | Model_2 | Chi_Square | P_Original | P_Adjusted |
| Radiologist | DeepSeek_a | 2.083 | 0.1489 (NS) | 1.0000 (NS) |
| Radiologist | DeepSeek_b | 0.1 | 0.7518 (NS) | 1.0000 (NS) |
| Radiologist | DeepSeek_c | 0.1 | 0.7518 (NS) | 1.0000 (NS) |
| Radiologist | Gemini_a | 2.769 | 0.0961 (NS) | 1.0000 (NS) |
| Radiologist | Gemini_b | 5.818 | 0.0159 (*) | 0.4124 (NS) |
| Radiologist | Gemini_c | 2.5 | 0.1138 (NS) | 1.0000 (NS) |
| Radiologist | GPT_a | 9.6 | 0.0019 (**) | 0.0525 (NS) |
| Radiologist | GPT_b | 4.923 | 0.0265 (*) | 0.6625 (NS) |
| Radiologist | GPT_c | 1.231 | 0.2673 (NS) | 1.0000 (NS) |
| DeepSeek_a | DeepSeek_b | 1.5 | 0.2207 (NS) | 1.0000 (NS) |
| DeepSeek_a | DeepSeek_c | 1.5 | 0.2207 (NS) | 1.0000 (NS) |
| DeepSeek_b | DeepSeek_c | 0 | 1.0000 (NS) | 1.0000 (NS) |
| Gemini_a | Gemini_b | 0.25 | 0.6171 (NS) | 1.0000 (NS) |
| Gemini_a | Gemini_c | 0 | 1.0000 (NS) | 1.0000 (NS) |
| Gemini_b | Gemini_c | 0.8 | 0.3711 (NS) | 1.0000 (NS) |
| GPT_a | GPT_b | 0.9 | 0.3428 (NS) | 1.0000 (NS) |
| GPT_a | GPT_c | 4.083 | 0.0433 (*) | 1.0000 (NS) |
| GPT_b | GPT_c | 1.5 | 0.2207 (NS) | 1.0000 (NS) |
| DeepSeek_a | Gemini_a | 0 | 1.0000 (NS) | 1.0000 (NS) |
| DeepSeek_a | GPT_a | 2.769 | 0.0961 (NS) | 1.0000 (NS) |
| Gemini_a | GPT_a | 3.125 | 0.0771 (NS) | 1.0000 (NS) |
| DeepSeek_b | Gemini_b | 4 | 0.0455 (*) | 1.0000 (NS) |
| DeepSeek_b | GPT_b | 3.273 | 0.0704 (NS) | 1.0000 (NS) |
| Gemini_b | GPT_b | 0 | 1.0000 (NS) | 1.0000 (NS) |
| DeepSeek_c | Gemini_c | 1.125 | 0.2888 (NS) | 1.0000 (NS) |
| DeepSeek_c | GPT_c | 0.364 | 0.5465 (NS) | 1.0000 (NS) |
| Gemini_c | GPT_c | 0 | 1.0000 (NS) | 1.0000 (NS) |
| **Liver_ disease diagnosis** | | | | |
| Model_1 | Model_2 | Chi_Square | P_Original | P_Adjusted |
| Radiologist | DeepSeek_a | 8.471 | 0.0036 (**) | 0.0902 (NS) |
| Radiologist | DeepSeek_b | 5.818 | 0.0159 (*) | 0.3489 (NS) |
| Radiologist | DeepSeek_c | 1.778 | 0.1824 (NS) | 1.0000 (NS) |
| Radiologist | Gemini_a | 8.471 | 0.0036 (**) | 0.0902 (NS) |
| Radiologist | Gemini_b | 6.75 | 0.0094 (**) | 0.2156 (NS) |
| Radiologist | Gemini_c | 3.125 | 0.0771 (NS) | 1.0000 (NS) |
| Radiologist | GPT_a | 12.19 | 0.0005 (***) | 0.0130 (*) |
| Radiologist | GPT_b | 8.643 | 0.0033 (**) | 0.0854 (NS) |
| Radiologist | GPT_c | 3.368 | 0.0665 (NS) | 1.0000 (NS) |
| DeepSeek_a | DeepSeek_b | 0.75 | 0.3865 (NS) | 1.0000 (NS) |
| DeepSeek_a | DeepSeek_c | 3.5 | 0.0614 (NS) | 1.0000 (NS) |
| DeepSeek_b | DeepSeek_c | 0.9 | 0.3428 (NS) | 1.0000 (NS) |
| Gemini_a | Gemini_b | 0.444 | 0.5050 (NS) | 1.0000 (NS) |
| Gemini_a | Gemini_c | 2.769 | 0.0961 (NS) | 1.0000 (NS) |
| Gemini_b | Gemini_c | 2.25 | 0.1336 (NS) | 1.0000 (NS) |
| GPT_a | GPT_b | 1.778 | 0.1824 (NS) | 1.0000 (NS) |
| GPT_a | GPT_c | 3.5 | 0.0614 (NS) | 1.0000 (NS) |
| GPT_b | GPT_c | 0.571 | 0.4497 (NS) | 1.0000 (NS) |
| DeepSeek_a | Gemini_a | 0 | 1.0000 (NS) | 1.0000 (NS) |
| DeepSeek_a | GPT_a | 0.5 | 0.4795 (NS) | 1.0000 (NS) |
| Gemini_a | GPT_a | 0.643 | 0.4227 (NS) | 1.0000 (NS) |
| DeepSeek_b | Gemini_b | 0 | 1.0000 (NS) | 1.0000 (NS) |
| DeepSeek_b | GPT_b | 0.444 | 0.5050 (NS) | 1.0000 (NS) |
| Gemini_b | GPT_b | 0.083 | 0.7728 (NS) | 1.0000 (NS) |
| DeepSeek_c | Gemini_c | 0 | 1.0000 (NS) | 1.0000 (NS) |
| DeepSeek_c | GPT_c | 0.562 | 0.4533 (NS) | 1.0000 (NS) |
| Gemini_c | GPT_c | 0.364 | 0.5465 (NS) | 1.0000 (NS) |
| **Lung_benign-malignant differentiation** | | | | |
| Model_1 | Model_2 | Chi_Square | P_Original | P_Adjusted |
| Radiologist | DeepSeek_a | 11.077 | 0.0009 (***) | 0.0227 (*) |
| Radiologist | DeepSeek_b | 7.111 | 0.0077 (**) | 0.1609 (NS) |
| Radiologist | DeepSeek_c | 6.667 | 0.0098 (**) | 0.1965 (NS) |
| Radiologist | Gemini_a | 8.1 | 0.0044 (**) | 0.0974 (NS) |
| Radiologist | Gemini_b | 5.143 | 0.0233 (*) | 0.4435 (NS) |
| Radiologist | Gemini_c | 12.071 | 0.0005 (***) | 0.0138 (*) |
| Radiologist | GPT_a | 9.091 | 0.0026 (**) | 0.0591 (NS) |
| Radiologist | GPT_b | 11.077 | 0.0009 (***) | 0.0227 (*) |
| Radiologist | GPT_c | 11.077 | 0.0009 (***) | 0.0227 (*) |
| DeepSeek_a | DeepSeek_b | 1.125 | 0.2888 (NS) | 1.0000 (NS) |
| DeepSeek_a | DeepSeek_c | 0.062 | 0.8026 (NS) | 1.0000 (NS) |
| DeepSeek_b | DeepSeek_c | 0.1 | 0.7518 (NS) | 1.0000 (NS) |
| Gemini_a | Gemini_b | 1.333 | 0.2482 (NS) | 1.0000 (NS) |
| Gemini_a | Gemini_c | 2.25 | 0.1336 (NS) | 1.0000 (NS) |
| Gemini_b | Gemini_c | 5.143 | 0.0233 (*) | 0.4435 (NS) |
| GPT_a | GPT_b | 0.167 | 0.6831 (NS) | 1.0000 (NS) |
| GPT_a | GPT_c | 0.25 | 0.6171 (NS) | 1.0000 (NS) |
| GPT_b | GPT_c | 0 | 1.0000 (NS) | 1.0000 (NS) |
| DeepSeek_a | Gemini_a | 0.364 | 0.5465 (NS) | 1.0000 (NS) |
| DeepSeek_a | GPT_a | 0.1 | 0.7518 (NS) | 1.0000 (NS) |
| Gemini_a | GPT_a | 0 | 1.0000 (NS) | 1.0000 (NS) |
| DeepSeek_b | Gemini_b | 0.125 | 0.7237 (NS) | 1.0000 (NS) |
| DeepSeek_b | GPT_b | 0.9 | 0.3428 (NS) | 1.0000 (NS) |
| Gemini_b | GPT_b | 3.125 | 0.0771 (NS) | 1.0000 (NS) |
| DeepSeek_c | Gemini_c | 0.267 | 0.6056 (NS) | 1.0000 (NS) |
| DeepSeek_c | GPT_c | 0.056 | 0.8137 (NS) | 1.0000 (NS) |
| Gemini_c | GPT_c | 0 | 1.0000 (NS) | 1.0000 (NS) |
| **Lung_ disease diagnosis** | | | | |
| Model_1 | Model_2 | Chi_Square | P_Original | P_Adjusted |
| Radiologist | DeepSeek_a | 15.059 | 0.0001 (***) | 0.0028 (**) |
| Radiologist | DeepSeek_b | 8.1 | 0.0044 (**) | 0.0885 (NS) |
| Radiologist | DeepSeek_c | 14.062 | 0.0002 (***) | 0.0046 (**) |
| Radiologist | Gemini_a | 9.091 | 0.0026 (**) | 0.0539 (NS) |
| Radiologist | Gemini_b | 6.125 | 0.0133 (*) | 0.2532 (NS) |
| Radiologist | Gemini_c | 13.067 | 0.0003 (***) | 0.0072 (**) |
| Radiologist | GPT_a | 10.083 | 0.0015 (**) | 0.0329 (*) |
| Radiologist | GPT_b | 14.062 | 0.0002 (***) | 0.0046 (**) |
| Radiologist | GPT_c | 12.071 | 0.0005 (***) | 0.0118 (*) |
| DeepSeek_a | DeepSeek_b | 3.273 | 0.0704 (NS) | 1.0000 (NS) |
| DeepSeek_a | DeepSeek_c | 0 | 1.0000 (NS) | 1.0000 (NS) |
| DeepSeek_b | DeepSeek_c | 3.125 | 0.0771 (NS) | 1.0000 (NS) |
| Gemini_a | Gemini_b | 1.333 | 0.2482 (NS) | 1.0000 (NS) |
| Gemini_a | Gemini_c | 1.5 | 0.2207 (NS) | 1.0000 (NS) |
| Gemini_b | Gemini_c | 5.143 | 0.0233 (*) | 0.3968 (NS) |
| GPT_a | GPT_b | 2.25 | 0.1336 (NS) | 1.0000 (NS) |
| GPT_a | GPT_c | 0.5 | 0.4795 (NS) | 1.0000 (NS) |
| GPT_b | GPT_c | 0.25 | 0.6171 (NS) | 1.0000 (NS) |
| DeepSeek_a | Gemini_a | 2.083 | 0.1489 (NS) | 1.0000 (NS) |
| DeepSeek_a | GPT_a | 1.455 | 0.2278 (NS) | 1.0000 (NS) |
| Gemini_a | GPT_a | 0 | 1.0000 (NS) | 1.0000 (NS) |
| DeepSeek_b | Gemini_b | 0.167 | 0.6831 (NS) | 1.0000 (NS) |
| DeepSeek_b | GPT_b | 2.5 | 0.1138 (NS) | 1.0000 (NS) |
| Gemini_b | GPT_b | 6.125 | 0.0133 (*) | 0.2532 (NS) |
| DeepSeek_c | Gemini_c | 0 | 1.0000 (NS) | 1.0000 (NS) |
| DeepSeek_c | GPT_c | 0.083 | 0.7728 (NS) | 1.0000 (NS) |
| Gemini_c | GPT_c | 0 | 1.0000 (NS) | 1.0000 (NS) |
| **Breast_classification** | | | | |
| Model_1 | Model_2 | Chi_Square | P_Original | P_Adjusted |
| Radiologist | DeepSeek_a | NA | NA | NA |
| Radiologist | DeepSeek_b | NA | NA | NA |
| Radiologist | DeepSeek_c | NA | NA | NA |
| Radiologist | Gemini_a | NA | NA | NA |
| Radiologist | Gemini_b | NA | NA | NA |
| Radiologist | Gemini_c | NA | NA | NA |
| Radiologist | GPT_a | NA | NA | NA |
| Radiologist | GPT_b | NA | NA | NA |
| Radiologist | GPT_c | NA | NA | NA |
| DeepSeek_a | DeepSeek_b | 0.129 | 0.7194 (NS) | 1.0000 (NS) |
| DeepSeek_a | DeepSeek_c | 0.833 | 0.3613 (NS) | 1.0000 (NS) |
| DeepSeek_b | DeepSeek_c | 0.19 | 0.6625 (NS) | 1.0000 (NS) |
| Gemini_a | Gemini_b | 1.389 | 0.2386 (NS) | 1.0000 (NS) |
| Gemini_a | Gemini_c | 0.267 | 0.6056 (NS) | 1.0000 (NS) |
| Gemini_b | Gemini_c | 0.444 | 0.5050 (NS) | 1.0000 (NS) |
| GPT_a | GPT_b | 0.75 | 0.3865 (NS) | 1.0000 (NS) |
| GPT_a | GPT_c | 1.455 | 0.2278 (NS) | 1.0000 (NS) |
| GPT_b | GPT_c | 0 | 1.0000 (NS) | 1.0000 (NS) |
| DeepSeek_a | Gemini_a | 0.148 | 0.7003 (NS) | 1.0000 (NS) |
| DeepSeek_a | GPT_a | 0.045 | 0.8312 (NS) | 1.0000 (NS) |
| Gemini_a | GPT_a | 0.593 | 0.4414 (NS) | 1.0000 (NS) |
| DeepSeek_b | Gemini_b | 4.654 | 0.0310 (*) | 0.4648 (NS) |
| DeepSeek_b | GPT_b | 0.138 | 0.7103 (NS) | 1.0000 (NS) |
| Gemini_b | GPT_b | 6.323 | 0.0119 (*) | 0.2146 (NS) |
| DeepSeek_c | Gemini_c | 6.05 | 0.0139 (*) | 0.2364 (NS) |
| DeepSeek_c | GPT_c | 0 | 1.0000 (NS) | 1.0000 (NS) |
| Gemini_c | GPT_c | 5.333 | 0.0209 (*) | 0.3347 (NS) |
| **Breast_benign-malignant differentiation** | | | | |
| Model_1 | Model_2 | Chi_Square | P_Original | P_Adjusted |
| Radiologist | DeepSeek_a | 7.682 | 0.0056 (**) | 0.1060 (NS) |
| Radiologist | DeepSeek_b | 6.05 | 0.0139 (*) | 0.2503 (NS) |
| Radiologist | DeepSeek_c | 8.45 | 0.0037 (**) | 0.0807 (NS) |
| Radiologist | Gemini_a | 8.522 | 0.0035 (**) | 0.0807 (NS) |
| Radiologist | Gemini_b | 8.522 | 0.0035 (**) | 0.0807 (NS) |
| Radiologist | Gemini_c | 8.522 | 0.0035 (**) | 0.0807 (NS) |
| Radiologist | GPT_a | 10.24 | 0.0014 (**) | 0.0330 (*) |
| Radiologist | GPT_b | 11.115 | 0.0009 (***) | 0.0214 (*) |
| Radiologist | GPT_c | 12.042 | 0.0005 (***) | 0.0135 (*) |
| DeepSeek_a | DeepSeek_b | 0.125 | 0.7237 (NS) | 1.0000 (NS) |
| DeepSeek_a | DeepSeek_c | 0 | 1.0000 (NS) | 1.0000 (NS) |
| DeepSeek_b | DeepSeek_c | 0.167 | 0.6831 (NS) | 1.0000 (NS) |
| Gemini_a | Gemini_b | 0 | 1.0000 (NS) | 1.0000 (NS) |
| Gemini_a | Gemini_c | 0 | 1.0000 (NS) | 1.0000 (NS) |
| Gemini_b | Gemini_c | 0 | 1.0000 (NS) | 1.0000 (NS) |
| GPT_a | GPT_b | 0 | 1.0000 (NS) | 1.0000 (NS) |
| GPT_a | GPT_c | 0 | 1.0000 (NS) | 1.0000 (NS) |
| GPT_b | GPT_c | 0 | 1.0000 (NS) | 1.0000 (NS) |
| DeepSeek_a | Gemini_a | 0 | 1.0000 (NS) | 1.0000 (NS) |
| DeepSeek_a | GPT_a | 0.364 | 0.5465 (NS) | 1.0000 (NS) |
| Gemini_a | GPT_a | 0.167 | 0.6831 (NS) | 1.0000 (NS) |
| DeepSeek_b | Gemini_b | 0.8 | 0.3711 (NS) | 1.0000 (NS) |
| DeepSeek_b | GPT_b | 2.083 | 0.1489 (NS) | 1.0000 (NS) |
| Gemini_b | GPT_b | 0.571 | 0.4497 (NS) | 1.0000 (NS) |
| DeepSeek_c | Gemini_c | 0 | 1.0000 (NS) | 1.0000 (NS) |
| DeepSeek_c | GPT_c | 0.643 | 0.4227 (NS) | 1.0000 (NS) |
| Gemini_c | GPT_c | 0.444 | 0.5050 (NS) | 1.0000 (NS) |
| **Breast_ disease diagnosis** | | | | |
| Model_1 | Model_2 | Chi_Square | P_Original | P_Adjusted |
| Radiologist | DeepSeek_a | 1.361 | 0.2433 (NS) | 1.0000 (NS) |
| Radiologist | DeepSeek_b | 0.658 | 0.4173 (NS) | 1.0000 (NS) |
| Radiologist | DeepSeek_c | 0.658 | 0.4173 (NS) | 1.0000 (NS) |
| Radiologist | Gemini_a | 0.39 | 0.5322 (NS) | 1.0000 (NS) |
| Radiologist | Gemini_b | 0.625 | 0.4292 (NS) | 1.0000 (NS) |
| Radiologist | Gemini_c | 0.457 | 0.4990 (NS) | 1.0000 (NS) |
| Radiologist | GPT_a | 0.837 | 0.3602 (NS) | 1.0000 (NS) |
| Radiologist | GPT_b | 0.34 | 0.5596 (NS) | 1.0000 (NS) |
| Radiologist | GPT_c | 0.205 | 0.6511 (NS) | 1.0000 (NS) |
| DeepSeek_a | DeepSeek_b | 0.083 | 0.7728 (NS) | 1.0000 (NS) |
| DeepSeek_a | DeepSeek_c | 0.083 | 0.7728 (NS) | 1.0000 (NS) |
| DeepSeek_b | DeepSeek_c | 0 | 1.0000 (NS) | 1.0000 (NS) |
| Gemini_a | Gemini_b | 0 | 1.0000 (NS) | 1.0000 (NS) |
| Gemini_a | Gemini_c | 0 | 1.0000 (NS) | 1.0000 (NS) |
| Gemini_b | Gemini_c | 0 | 1.0000 (NS) | 1.0000 (NS) |
| GPT_a | GPT_b | 0.125 | 0.7237 (NS) | 1.0000 (NS) |
| GPT_a | GPT_c | 0.444 | 0.5050 (NS) | 1.0000 (NS) |
| GPT_b | GPT_c | 0 | 1.0000 (NS) | 1.0000 (NS) |
| DeepSeek_a | Gemini_a | 0.308 | 0.5791 (NS) | 1.0000 (NS) |
| DeepSeek_a | GPT_a | 0 | 1.0000 (NS) | 1.0000 (NS) |
| Gemini_a | GPT_a | 0.1 | 0.7518 (NS) | 1.0000 (NS) |
| DeepSeek_b | Gemini_b | 0 | 1.0000 (NS) | 1.0000 (NS) |
| DeepSeek_b | GPT_b | 0 | 1.0000 (NS) | 1.0000 (NS) |
| Gemini_b | GPT_b | 0 | 1.0000 (NS) | 1.0000 (NS) |
| DeepSeek_c | Gemini_c | 0 | 1.0000 (NS) | 1.0000 (NS) |
| DeepSeek_c | GPT_c | 0.05 | 0.8231 (NS) | 1.0000 (NS) |
| Gemini_c | GPT_c | 0 | 1.0000 (NS) | 1.0000 (NS) |
